# Supplementary material for: Level of overweight and obesity surpassed underweight among women in 40 low and middle-income countries: Findings from a multilevel multinomial analysis of population survey data
Source: PLoS One. 2025 Mar 26;20(3):e0320095. doi: 10.1371/journal.pone.0320095 (PMC11940715; doi:10.1371/journal.pone.0320095)
Supplement: Attachment — Submitted filename: Response to Reviewers.pdf [file pone.0320095.s006.pdf]

Dear Academic Editor,

Thank you again for the opportunity to revise our manuscript "***Level of overweight and obesity surpassed underweight among women in 40 low and middle-income countries: findings from a multilevel multinomial analysis of population survey data (ID: PONE-D-24-26578R1)***". We have addressed the concerns raised using a point-by-point response as stated below. The amendments made to the manuscript have been presented using track change in the attachment titled "Revised manuscript with track changes".

Response to Editor's comments

Comment 1: The Methods Section casts doubts on the estimate: (i) It appears that authors are referring to many dependent variables yet there is only one (BMI, categorized); (ii) It is unclear in this section that overweight and obesity are treated as one category; (iii) while authors are doing multilevel modeling, it is unclear in this section what are Levl-1 and Level-2 variables. More importantly, it is unclear how Level-2 variables (also referred to as community variables) were constructed.

Response 1: Thank you for your important concern and sorry for the editorial errors. We have amended the description of the dependent variable (BMI) and clarified that overweight and obesity were treated as one category. Additionally, we have specified level-1 and level-2 variables in the revised manuscript.

Comment 2: It is unclear that analyses were performed on sub-samples of women of reproductive ages in all surveys since DHSs don't collect weight and height for all women to compute BMI. As such, authors took the freedom to DROP cases with missing information on BMI. This is a "big mistake from the sampling design point of view" because it will produce biased estimates. (see https://stats.oarc.ucla.edu/stata/faq/how-can-i-analyze-a-subpopulation-of-my-survey-data-in stata/#:~:text=As%20stated%20in%20the%20Stata,are%20excluded%20from%20the%20analysis.)

Response 2: Thank you very much for pointing out this. We have stated that the analysis was performed on the sub-sample of women of reproductive age in all surveys. Regarding missing

information, we agree with your concern that missing data can result in a biased estimate if not properly handled. For this reason, we have handled the missing data based on DHS guidelines (i.e. "women who were not weighed and measured and women whose values for weight and height were not recorded are excluded from both the denominator and the numerators.") (see https://www.dhsprogram.com/pubs/pdf/DHSG1/Guide_to_DHS_Statistics_DHS-8.pdf on Page 11.10). Additionally, we have also acknowledged the drawback of missing data in the limitation section of the revision.

Comment 3: Finally, the Discussion Section also needs refinement because it is redundant with findings. The authors need to engage in discussion with previous studies on the same topic while highlighting regional differences and implications for future health interventions.
Response 3: Thank you for your important recommendation. We have removed redundant results throughout the discussion section as per your suggestion.

Response to comments in the annotated manuscript
Comment 1: Line 22-24: A methodological challenge arises when pooling data. I will look into the Methods Section but this needs to be clear on how Complex Survey Design was implemented in pooled data.
Response 1: Thank you for your concern. As we have stated in the data management and analysis sub-heading of the method section that we have used pooled/appended demographic and health survey data from 44 countries for the current analysis.

Comment 2: Line 26: abnormal nutritional indices (This means what?)
Response 2: Thank you. It was to indicate malnutrition (i.e. underweight and overweight and obesity). As a result, we have specified this concern in the revised manuscript.

Comment 3: Line 30-31: Now and again, this is the pitfall I usually see in pooled data since LMICs are not a homogeneous group on the one hand, and context matters on the other hand. Is this finding informative?
Response 3: Thank you very much for your concern. We have carried out a subgroup analysis to show regional differences in the revised manuscript. We also agree that pooling data from

different countries has its drawbacks since the countries are not homogeneous and survey years are different. For this reason, we have considered these issues under the limitation section.

Comment 4: Line 33: women of middle and upper-class families?

Response 4: Thank you and sorry for the grammar error. We have changed this phrase into “women of families with middle and rich wealth indexes” accordingly in the revised manuscript.

Comment 5: Line 34: From primary to higher education? What about those with secondary education?

Response 5: Thank you for your question. For the analysis purpose, we have initially categorized educational status as no formal education, primary, secondary, and higher education. However, when we fit the regression model, the model omits the estimation of the relative risk ratio for the category “secondary education”. As a result, we recategorized this variable as “no formal education”, “primary education”, and “higher education (i.e. secondary + higher)”.

Comment 6: Line 37: You need to clarify the difference between middle- and upper-income families (By the way, income is not captured in DHS), and rich households.

Response 6: Thank you for your suggestion. In DHS, the household wealth index is categorized as poorest, poorer, middle, richer, and richest. For the analysis purpose, we have recategorized it as “poor”, “middle”, and “rich” based on the literature. Based on your suggestion, we changed these phrases to their correct DHS categories.

Comment 7: Line 43: modifiable risk factors of what?

Response 7: Thank you. It is just to indicate modifiable risk factors of both undernutrition and overnutrition. We have clarified this concern in the revised manuscript.

Comment 8: Line 62-64: You have furthermore and additionally in the same paragraph?

Response 8: Thank you very much. We have addressed this concern accordingly in the revised manuscript.

Comment 9: Line 72-74: You cannot list figures like this. Rewrite the sentence... using for instance: ranging from .... to ....

Response 9: Thank you for your interesting suggestion. We have revised this sentence as per your recommendation.

Comment 10: Line 77-80: same as above comment.

Response 10: Thank you for your interesting suggestion. We have revised this sentence as per your recommendation.

Comment 11: Line 103: Again, this poses a methodological concern that needs further scrutiny for reported findings to be trusted. 1) In the DHS, not all women in surveys were measured (weight, height) to compute BMI. As such, only a sub-sample of women has valid information on BMI. 2) How the complex survey design of DHS was treated in this case?

Response 11: Thank you for your important concern. Indeed, all women in the survey do not have BMI data (missing or not measured). For this reason, we have excluded cases with missing data based on DHS guidelines for handling missing data, and this issue was also acknowledged in the limitation section. Regarding the treatment complex survey design, we have used a multilevel model, which accounts for the hierarchical nature of the complex survey.

Comment 12: Line 107: This is not TRUE. The dependent variable is only one: BMI. Analytically, it was grouped into four categories: 1) underweight; 2) normal weight; 3) overweight; and 4) obese. Now, in many developing countries, obesity might represent a marginal percentage, which could lead to unstable statistical models. Please clarify your line of thought.

Response 12: Thank you very much for pointing out this important issue. As per your recommendation, we have clarified the dependent variable as BMI, with its respective categories (underweight, Normal, and overweight and obesity). We have also stated that overweight and obesity are treated as one category in the revised manuscript.

Comment 13: Line 112- Since you applied multilevel models, it helps readers if variables are grouped for each level

Response 13: Thank you for your suggestion. We have specified level-1 and level-2 variables in the revision.

Comment 14: Line 128-129: This is a serious methodological pitfall since it alters the survey design and produces "flawed" estimates

Response 14: Thank you very much for pointing out this. We have stated that the analysis was performed on the sub-sample of women of reproductive age from all surveys in the revision. Regarding missing information, we agree with your concern that missing data can result in a biased estimate if not properly handled. For this reason, we have handled the missing data based on DHS guidelines (i.e. "women who were not weighed and measured and women whose values for weight and height were not recorded are excluded from both the denominator and the numerators.") (see https://www.dhsprogram.com/pubs/pdf/DHSG1/Guide_to_DHS_Statistics_DHS-8.pdf on Page 11.10). Additionally, we have also acknowledged the drawback of missing data in the limitation section of the revision.

Comment 15: Line 132: Level 1? Level 2? In short, how many LEVELS?

Response 15: Thank you very much. We have specified level-1 and level-2 variables under the "Independent variables" subsection of the revised manuscript.

Comment 16: Line 46: unknown as presented

Response 16: Thank you for your concern. We have replaced this with level-1 variables as per your suggestion in the "Independent variables" section.

Comment 17: Line 181: full term of WRA.

Response 17: Thank you. We have provided the extended form of WRA (i.e. women of reproductive age) in the revised result section.

Comment 18: Line 192: Reproductive and health-related characteristics. This appears for the first time.

Response 18: Thank you for your concern. For the descriptive purpose, we have separately presented the characteristics of the study participants as "Sociodemographic and reproductive-related characteristics".

Comment 19: Line 211: In Figures 1-2, countries are thrown as they belonged to the same geographic region. Groupings need to be done.

Response 19: Thank you for your interesting comment. We have carried out subgroup analysis to show country-, regional, and overall estimates of underweight and overweight and obesity based on your suggestion. As a result, we have changed Figures 1 and 2 based on the reanalysis results.

Comment 20: Line 214: Finally, this contradicts what is said in the Methods Section regarding BMI (4 categories) but findings use only categories

Response 20: Thank you for your concern. We have modified the definition and categories of the outcome variable and stated that overweight and obesity are treated as one category as per our suggestion.

Comment 21: Line 266: As it stands now, the discussion is flawed: It is mostly a repetition of findings.

Response 21: Thank you for your important recommendation. We have removed redundant results throughout the discussion section as per your suggestion.

Comment 22: Line 267: It should be clear throughout the manuscript that overweight and obesity are treated as one category.

Response 22: Thank you for your suggestion. We have clarified that overweight and obesity were treated as one category throughout the revised manuscript.

Comment 23: Line 292: what does middle class mean? You use interchangeably income, class, households???? WHY, explain!

Response 23: Thank you very much and sorry for the editorial error. We have consistently used the appropriate terms for the household wealth index throughout the revised manuscript.

Comment 24: Line 313: what about women with secondary education

Response 24: Thank you for your question. For the analysis purpose, we have initially categorized educational status as no formal education, primary, secondary, and higher education. However, when we fit the regression model, the model omits the estimation of the relative risk ratio for the category "secondary education". As a result, we re-categorized this variable as "no formal education", "primary education", and "higher education (i.e. secondary + higher)".

Comment 25: Line 318-319: I expect that higher education leads to wealth, but at the same time leads to sedentary lifestyles (always in cars, with maids in houses, low levels of physical activity).

Response 25: Thank you for your interesting comment. We have added this explanation to the revised manuscript.

Comment 26: Line 346: Please be specific

Response 26: Thank you. We have amended this concern throughout the revised manuscript.

Response to Reviewers' comments

Reviewer #1: The manuscript entitled "Level of overweight and obesity surpassed underweight among women in 40 low and middle-income countries: findings from a multilevel multinomial analysis of population survey data". I appreciate the authors' efforts. The authors manage to respond to all the issues perfectly.

Response: Thank you for your appreciation and for accepting our manuscript in its current form.

Reviewer #2: The authors addressed all the comments and concerns raised in the first revision. I have no further comment.

Response: Thank you for accepting our manuscript in its current form.
