# Supplementary material for: Level of overweight and obesity surpassed underweight among women in 40 low and middle-income countries: Findings from a multilevel multinomial analysis of population survey data
Source: PLoS One. 2025 Mar 26;20(3):e0320095. doi: 10.1371/journal.pone.0320095 (PMC11940715; doi:10.1371/journal.pone.0320095)
Supplement: Attachment — Submitted filename: PONE-D-24-26578_R2.pdf [file pone.0320095.s005.pdf]

PLOS ONE

Level of overweight and obesity surpassed underweight among women in 40 low and middle-income countries: findings from a multilevel multinomial analysis of population survey data

--Manuscript Draft--

Manuscript Number:	PONE-D-24-26578R2
Article Type:	Research Article
Full Title:	Level of overweight and obesity surpassed underweight among women in 40 low and middle-income countries: findings from a multilevel multinomial analysis of population survey data
Short Title:	Level of overweight and obesity surpassed underweight among women in 40 low- and middle income countries
Corresponding Author:	Kusse Urmale Mare, MSc<br>Samara University<br>Semera-Logiya city administration, Afar regional state ETHIOPIA
Keywords:	Low and middle-income countries; Underweight; Overweight; obesity; Demographic and Health Survey; Determinants; Multilevel multinomial analysis; Women of Reproductive age.
Abstract:	Background: Despite continued global and local initiatives to address nutritional problems, low- and middle-income countries are facing both malnutrition and non-communicable diseases, with about 80% of non-communicable disease-related deaths. There is a dearth of recent evidence on the extent and determinants of underweight, overweight, and obesity in this region, which is essential for guiding intervention programs. Thus, this study intended to provide insights into the current level of malnutrition among women of reproductive age in low- and middle-income countries.<br>Methods: A secondary analysis of Demographic and Health Survey data from 40 low- and middle-income countries was performed using a weighted sample of 1,044,340 women of reproductive age. Forest plots and line graph were used to present the pooled and regional estimates of underweight and overweight and obesity. A multilevel multinomial logistic regression models were fitted to identify determinants of malnutrition and models were compared based on the deviance and log-likelihood values. In the final model, statistical significance was determined using an adjusted relative risk ratio with the corresponding 95% confidence interval.<br>Results: The overall prevalence of obesity and overweight was 26.8% (95% CI: 26.7%-26.9%), with the highest rate in Jordan (67.2%) and lowest in Burundi (9.5%). Additionally, the level of underweight was found to be 15.6% [95% CI: 15.5%-15.7%], ranging from 1.3% in Jordan to 25.4% in Timor-Leste. Women of families with middle and rich wealth indexes, those aged 25-34 and 35-49, contraceptive users, those with primary and higher education, and urban residents were more likely to be overweight or obese. In contrast, the results showed a lower likelihood of undernutrition among women in the households with middle [ARRR (95% CI): 0.80 (0.78-0.83)] and rich wealth indexes [ARRR (95% CI): 0.69 (0.67-0.71)], those with primary [ARRR (95% CI): 0.68 (0.66-0.71)] and higher education [ARRR (95% CI): 0.65 (0.63-0.67)], and women with media access [ARRR (95% CI): 0.79 (0.77-0.81)].<br>Conclusion: Over a quarter of women of reproductive age in LMICs were overweight or obese, with underweight affecting 15.6%. It was also found that the levels of overweight and obesity were higher than the rate for underweight, with wide variations across the countries. Thus, efforts to improve the modifiable risks can have a positive impact on reducing undernutrition and over-nutrition. Given the considerable variations between countries and survey periods, nutrition programs should also be tailored to the specific needs and cultural contexts of different regions within the study settings. Furthermore, the findings suggest the need for regular monitoring and evaluation of the existing nutritional interventions to assess their effectiveness.
Order of Authors:	Kusse Urmale Mare, MSc
	Kebede Gemeda Sabo, MSc

Powered by Editorial Manager® and ProduXion Manager® from Aries Systems Corporation

	Beriso Furo Wengoro, MSc
	Begetayinoral Kussia Lahole, MSc
**Opposed Reviewers:**	
**Response to Reviewers:**	Dear Academic Editor,<br>Thank you again for the opportunity to revise our manuscript “Level of overweight and obesity surpassed underweight among women in 40 low and middle-income countries: findings from a multilevel multinomial analysis of population survey data (ID: PONE-D-24-26578R1)“. We have addressed the concerns raised using a point-by-point response as stated below. The amendments made to the manuscript have been presented using track change in the attachment titled “Revised manuscript with track changes”.<br><br>Response to Editor’s comments<br>Comment 1: The Methods Section casts doubts on the estimate: (i) It appears that authors are referring to many dependent variables yet there is only one (BMI, categorized); (ii) It is unclear in this section that overweight and obesity are treated as one category; (iii) while authors are doing multilevel modeling, it is unclear in this section what are Levl-1 and Level-2 variables. More importantly, it is unclear how Level-2 variables (also referred to as community variables) were constructed.<br>Response 1: Thank you for your important concern and sorry for the editorial errors. We have amended the description of the dependent variable (BMI) and clarified that overweight and obesity were treated as one category. Additionally, we have specified level-1 and level-2 variables in the revised manuscript.<br>Comment 2: It is unclear that analyses were performed on sub-samples of women of reproductive ages in all surveys since DHSs don't collect weight and height for all women to compute BMI. As such, authors took the freedom to DROP cases with missing information on BMI. This is a "big mistake from the sampling design point of view" because it will produce biased estimates. (see https://stats.oarc.ucla.edu/stata/faq/how-can-i-analyze-a-subpopulation-of-my-survey-data-in stata/#:~:text=As%20stated%20in%20the%20Stata,are%20excluded%20from%20the%20analysis.)<br>Response 2: Thank you very much for pointing out this. We have stated that the analysis was performed on the sub-sample of women of reproductive age in all surveys. Regarding missing information, we agree with your concern that missing data can result in a biased estimate if not properly handled. For this reason, we have handled the missing data based on DHS guidelines (i.e. “women who were not weighed and measured and women whose values for weight and height were not recorded are excluded from both the denominator and the numerators.”) (see https://www.dhsprogram.com/pubs/pdf/DHSG1/Guide_to_DHS_Statistics_DHS-8.pdf on Page 11.10). Additionally, we have also acknowledged the drawback of missing data in the limitation section of the revision.<br>Comment 3: Finally, the Discussion Section also needs refinement because it is redundant with findings. The authors need to engage in discussion with previous studies on the same topic while highlighting regional differences and implications for future health interventions.<br>Response 3: Thank you for your important recommendation. We have removed redundant results throughout the discussion section as per your suggestion.<br><br>Response to comments in the annotated manuscript<br>Comment 1: Line 22-24: A methodological challenge arises when pooling data. I will look into the Methods Section but this needs to be clear on how Complex Survey Design was implemented in pooled data.<br>Response 1: Thank you for your concern. As we have stated in the data management and analysis sub-heading of the method section that we have used pooled/appended demographic and health survey data from 44 countries for the current analysis.<br>Comment 2: Line 26: abnormal nutritional indices (This means what?)<br>Response 2: Thank you. It was to indicate malnutrition (i.e. underweight and overweight and obesity). As a result, we have specified this concern in the revised manuscript.<br>Comment 3: Line 30-31: Now and again, this is the pitfall I usually see in pooled data since LMICs are not a homogeneous group on the one hand, and context matters on

	the other hand. Is this finding informative?<br>Response 3: Thank you very much for your concern. We have carried out a subgroup analysis to show regional differences in the revised manuscript. We also agree that pooling data from different countries has its drawbacks since the countries are not homogeneous and survey years are different. For this reason, we have considered these issues under the limitation section.<br>Comment 4: Line 33: women of middle and upper-class families?<br>Response 4: Thank you and sorry for the grammar error. We have changed this phrase into “women of families with middle and rich wealth indexes” accordingly in the revised manuscript.<br>Comment 5: Line 34: From primary to higher education? What about those with secondary education?<br>Response 5: Thank you for your question. For the analysis purpose, we have initially categorized educational status as no formal education, primary, secondary, and higher education. However, when we fit the regression model, the model omits the estimation of the relative risk ratio for the category “secondary education”. As a result, we recategorized this variable as “no formal education”, “primary education”, and “higher education (i.e. secondary + higher)”.<br>Comment 6: Line 37: You need to clarify the difference between middle- and upper-income families (By the way, income is not captured in DHS), and rich households.<br>Response 6: Thank you for your suggestion. In DHS, the household wealth index is categorized as poorest, poorer, middle, richer, and richest. For the analysis purpose, we have recategorized it as “poor”, “middle”, and “rich” based on the literature. Based on your suggestion, we changed these phrases to their correct DHS categories.<br>Comment 7: Line 43: modifiable risk factors of what?<br>Response 7: Thank you. It is just to indicate modifiable risk factors of both undernutrition and overnutrition. We have clarified this concern in the revised manuscript.<br>Comment 8: Line 62-64: You have furthermore and additionally in the same paragraph?<br>Response 8: Thank you very much. We have addressed this concern accordingly in the revised manuscript.<br>Comment 9: Line 72-74: You cannot list figures like this. Rewrite the sentence... using for instance: ranging from .... to ....<br>Response 9: Thank you for your interesting suggestion. We have revised this sentence as per your recommendation.<br>Comment 10: Line 77-80: same as above comment.<br>Response 10: Thank you for your interesting suggestion. We have revised this sentence as per your recommendation.<br>Comment 11: Line 103: Again, this poses a methodological concern that needs further scrutiny for reported findings to be trusted. 1) In the DHS, not all women in surveys were measured (weight, height) to compute BMI. As such, only a sub-sample of women has valid information on BMI. 2) How the complex survey design of DHS was treated in this case?<br>Response 11: Thank you for your important concern. Indeed, all women in the survey do not have BMI data (missing or not measured). For this reason, we have excluded cases with missing data based on DHS guidelines for handling missing data, and this issue was also acknowledged in the limitation section. Regarding the treatment complex survey design, we have used a multilevel model, which accounts for the hierarchical nature of the complex survey.<br>Comment 12: Line 107: This is not TRUE. The dependent variable is only one: BMI. Analytically, it was grouped into four categories: 1) underweight; 2) normal weight; 3) overweight; and 4) obese. Now, in many developing countries, obesity might represent a marginal percentage, which could lead to unstable statistical models. Please clarify your line of thought.<br>Response 12: Thank you very much for pointing out this important issue. As per your recommendation, we have clarified the dependent variable as BMI, with its respective categories (underweight, Normal, and overweight and obesity). We have also stated that overweight and obesity are treated as one category in the revised manuscript.<br>Comment 13: Line 112- Since you applied multilevel models, it helps readers if variables are grouped for each level<br>Response 13: Thank you for your suggestion. We have specified level-1 and level-2 variables in the revision.<br>Comment 14: Line 128-129: This is a serious methodological pitfall since it alters the

	survey design and produces "flawed" estimates<br>Response 14: Thank you very much for pointing out this. We have stated that the analysis was performed on the sub-sample of women of reproductive age from all surveys in the revision. Regarding missing information, we agree with your concern that missing data can result in a biased estimate if not properly handled. For this reason, we have handled the missing data based on DHS guidelines (i.e. “women who were not weighed and measured and women whose values for weight and height were not recorded are excluded from both the denominator and the numerators.”) (see https://www.dhsprogram.com/pubs/pdf/DHSG1/Guide_to_DHS_Statistics_DHS-8.pdf on Page 11.10). Additionally, we have also acknowledged the drawback of missing data in the limitation section of the revision.<br>Comment 15: Line 132: Level 1? Level 2? In short, how many LEVELS?<br>Response 15: Thank you very much. We have specified level-1 and level-2 variables under the “Independent variables” subsection of the revised manuscript.<br>Comment 16: Line 46: unknown as presented<br>Response 16: Thank you for your concern. We have replaced this with level-1 variables as per your suggestion in the “Independent variables” section.<br>Comment 17: Line 181: full term of WRA.<br>Response 17: Thank you. We have provided the extended form of WRA (i.e. women of reproductive age) in the revised result section.<br>Comment 18: Line 192: Reproductive and health-related characteristics. This appears for the first time.<br>Response 18: Thank you for your concern. For the descriptive purpose, we have separately presented the characteristics of the study participants as “Sociodemographic and reproductive-related characteristics”.<br>Comment 19: Line 211: In Figures 1-2, countries are thrown as they belonged to the same geographic region. Groupings need to be done.<br>Response 19: Thank you for your interesting comment. We have carried out subgroup analysis to show country-, regional, and overall estimates of underweight and overweight and obesity based on your suggestion. As a result, we have changed Figures 1 and 2 based on the reanalysis results.<br>Comment 20: Line 214: Finally, this contradicts what is said in the Methods Section regarding BMI (4 categories) but findings use only categories<br>Response 20: Thank you for your concern. We have modified the definition and categories of the outcome variable and stated that overweight and obesity are treated as one category as per our suggestion.<br>Comment 21: Line 266: As it stands now, the discussion is flawed: It is mostly a repetition of findings.<br>Response 21: Thank you for your important recommendation. We have removed redundant results throughout the discussion section as per your suggestion.<br>Comment 22: Line 267: It should be clear throughout the manuscript that overweight and obesity are treated as one category.<br>Response 22: Thank you for your suggestion. We have clarified that overweight and obesity were treated as one category throughout the revised manuscript.<br>Comment 23: Line 292: what does middle class mean? You use interchangeably income, class, households???? WHY, explain!<br>Response 23: Thank you very much and sorry for the editorial error. We have consistently used the appropriate terms for the household wealth index throughout the revised manuscript.<br>Comment 24: Line 313: what about women with secondary education<br>Response 24: Thank you for your question. For the analysis purpose, we have initially categorized educational status as no formal education, primary, secondary, and higher education. However, when we fit the regression model, the model omits the estimation of the relative risk ratio for the category “secondary education”. As a result, we re-categorized this variable as “no formal education”, “primary education”, and “higher education (i.e. secondary + higher)”.<br>Comment 25: Line 318-319: I expect that higher education leads to wealth, but at the same time leads to sedentary lifestyles (always in cars, with maids in houses, low levels of physical activity).<br>Response 25: Thank you for your interesting comment. We have added this explanation to the revised manuscript.<br>Comment 26: Line 346: Please be specific<br>Response 26: Thank you. We have amended this concern throughout the revised manuscript.

Powered by Editorial Manager® and ProduXion Manager® from Aries Systems Corporation

	Response to Reviewers' comments<br>Reviewer #1: The manuscript entitled "Level of overweight and obesity surpassed underweight among women in 40 low and middle-income countries: findings from a multilevel multinomial analysis of population survey data". I appreciate the authors' efforts. The authors manage to respond to all the issues perfectly.<br>Response: Thank you for your appreciation and for accepting our manuscript in its current form.<br>Reviewer #2: The authors addressed all the comments and concerns raised in the first revision. I have no further comment.<br>Response: Thank you for accepting our manuscript in its current form.
**Additional Information:**	
**Question**	**Response**
**Financial Disclosure**<br><br>Enter a financial disclosure statement that describes the sources of funding for the work included in this submission. Review the submission guidelines for detailed requirements. View published research articles from *PLOS ONE* for specific examples.<br><br>This statement is required for submission and **will appear in the published article** if the submission is accepted. Please make sure it is accurate.<br><br>**Funded studies**<br>Enter a statement with the following details:<br>• Initials of the authors who received each award<br>• Grant numbers awarded to each author<br>• The full name of each funder<br>• URL of each funder website<br>• Did the sponsors or funders play any role in the study design, data collection and analysis, decision to publish, or preparation of the manuscript?<br><br>Did you receive funding for this work?	The author(s) received no specific funding for this work.
**Competing Interests**<br><br>Use the instructions below to enter a competing interest statement for this submission. On behalf of all authors, disclose any competing interests that could be perceived to bias this work—acknowledging all financial support and any other relevant financial or non-	The authors have declared that no competing interests exist.

Powered by Editorial Manager® and ProduXion Manager® from Aries Systems Corporation

financial competing interests.<br><br>This statement is required for submission and **will appear in the published article** if the submission is accepted. Please make sure it is accurate and that any funding sources listed in your Funding Information later in the submission form are also declared in your Financial Disclosure statement.<br><br>View published research articles from *PLOS ONE* for specific examples.<br><br>**NO authors have competing interests**<br><br>Enter: *The authors have declared that no competing interests exist.*<br><br>**Authors with competing interests**<br><br>Enter competing interest details beginning with this statement:<br><br>*I have read the journal's policy and the authors of this manuscript have the following competing interests: [insert competing interests here]*<br><br>* typeset	
**Ethics Statement**<br><br>Enter an ethics statement for this submission. This statement is required if the study involved:<br><br>• Human participants<br>• Human specimens or tissue<br>• Vertebrate animals or cephalopods<br>• Vertebrate embryos or tissues<br>• Field research<br><br>Write "N/A" if the submission does not require an ethics statement.<br><br>General guidance is provided below. Consult the submission guidelines for	Since we used secondary data, permission to access the data was granted from the Measure DHS official Database via an online request at http://www.dhsprogram.com. The survey procedures were also approved by the Institutional Review Board of the host country and ICF International. Additionally, written informed consent was initially obtained from the study participants during the collection of the survey data.

Powered by Editorial Manager® and ProduXion Manager® from Aries Systems Corporation

detailed instructions. **Make sure that all information entered here is included in the Methods section of the manuscript.**<br><br>**Format for specific study types**<br><br>**Human Subject Research (involving human participants and/or tissue)**<br>• Give the name of the institutional review board or ethics committee that approved the study<br>• Include the approval number and/or a statement indicating approval of this research<br>• Indicate the form of consent obtained (written/oral) or the reason that consent was not obtained (e.g. the data were analyzed anonymously)<br><br>**Animal Research (involving vertebrate animals, embryos or tissues)**<br>• Provide the name of the Institutional Animal Care and Use Committee (IACUC) or other relevant ethics board that reviewed the study protocol, and indicate whether they approved this research or granted a formal waiver of ethical approval<br>• Include an approval number if one was obtained<br>• If the study involved *non-human primates*, add *additional details* about animal welfare and steps taken to ameliorate suffering<br>• If anesthesia, euthanasia, or any kind of animal sacrifice is part of the study, include briefly which substances and/or methods were applied<br><br>**Field Research**<br><br>Include the following details if this study involves the collection of plant, animal, or other materials from a natural setting:<br>• Field permit number<br>• Name of the institution or relevant body that granted permission	
**Data Availability**<br><br>Authors are required to make all data underlying the findings described fully available, without restriction, and from the time of publication. PLOS allows rare	Yes - all data are fully available without restriction

exceptions to address legal and ethical concerns. See the PLOS Data Policy and FAQ for detailed information.<br><br>A Data Availability Statement describing where the data can be found is required at submission. Your answers to this question constitute the Data Availability Statement and **will be published in the article**, if accepted.<br><br>**Important:** Stating 'data available on request from the author' is not sufficient. If your data are only available upon request, select 'No' for the first question and explain your exceptional situation in the text box.<br><br>Do the authors confirm that all data underlying the findings described in their manuscript are fully available without restriction?	
**Describe where the data may be found in full sentences. If you are copying our sample text, replace any instances of XXX with the appropriate details.**<br><br>• If the data are **held or will be held in a public repository**, include URLs, accession numbers or DOIs. If this information will only be available after acceptance, indicate this by ticking the box below. For example: *All XXX files are available from the XXX database (accession number(s) XXX, XXX.*).<br>• If the data are all contained **within the manuscript and/or Supporting Information files**, enter the following: *All relevant data are within the manuscript and its Supporting Information files.*<br>• If neither of these applies but you are able to provide **details of access elsewhere**, with or without limitations, please do so. For example:<br><br>*Data cannot be shared publicly because of [XXX]. Data are available from the XXX Institutional Data Access / Ethics Committee (contact via XXX) for researchers who meet the criteria for*	The data used and analyzed in this study can be accessed from the DHS website (https://dhsprogram.com/data/dataset_admin/index.cfm).

Powered by Editorial Manager® and ProduXion Manager® from Aries Systems Corporation

access to confidential data.<br><br>*The data underlying the results presented in the study are available from (include the name of the third party and contact information or URL).*<br>• This text is appropriate if the data are owned by a third party and authors do not have permission to share the data.<br><br>* typeset	
Additional data availability information:	

Dear Academic Editor,

Accompanying this letter, you will find our manuscript entitled ***"Level of overweight and obesity surpassed underweight among women in 40 low and middle-income countries: findings from a multilevel multinomial analysis of population survey data"*** submitted by Mr. Kusse Urmale to be considered for publication as an original research article in PLOS ONE.

Despite continued global and local initiatives to address nutritional problems, low- and middle-income countries are facing both malnutrition and non-communicable diseases, with about 80% of NCD-related deaths. There is a dearth of recent evidence on the extent and determinants of underweight, overweight, and obesity in this region, which is essential for guiding intervention programs. A secondary analysis of DHS data from 40 LMICs was done on a weighted sample of 1,044,340 women was considered for analysis. A multilevel multinomial logistic regression model was fitted to identify determinants of abnormal nutritional indices.

The overall prevalence of obesity and overweight was 26.8% (95% IC: 26.7%-26.9%), with the highest rate in Jordan (67.2%) and lowest in Burundi (9.5%). Additionally, the level of underweight was found to be 15.6% [95% CI: 15.5%-15.7%], ranging from 1.3% in Jordan to 25.4% in Timor-Leste. Our analysis indicated the bidirectional influence of household wealth, women's education, age, employment status, contraceptive use, region, place of residence, and media access on the risk of being underweight, overweight, and obese. We believe these findings will contribute as input for the efforts being undertaken to improve maternal nutrition and will be of interest to the reader of your journal.

We declare that this research is original, has not been published before, and is not currently being considered for publication elsewhere.

We know of no conflicts of interest associated with the publication, and there is no financial support for this work. As a corresponding author, I confirm that the manuscript has been read and approved for submission by all authors.

We look forward to your consideration of this manuscript in your prestigious journal.

Best regards,

Kusse Urmale Mare

Email: kussesinb@gmail.com

Revised Manuscript
Click here to access/download;Manuscript;Revised manuscript R2.docx

Level of overweight and obesity surpassed underweight among women in 40 low and middle-income countries: findings from a multilevel multinomial analysis of population survey data

Kusse Urmale Mare[1]*, Kebede Gemeda Sabo[1], Beriso Furo Wengoro[2], Begetayinoral Kussia Lahole[3]

Affiliations

[1]Department of Nursing, College of Medicine and Health Sciences, Samara University, Samara, Ethiopia

[2]Department of Biomedical Sciences, College of Medicine and Health Sciences, Samara University, Samara, Ethiopia

[3]Department of Midwifery, College of Medicine and Health Sciences, Arba Minch University, Arba Minch, Ethiopia

***Corresponding author**: Kusse Urmale Mare; Email: kussesinbo@gmail.com

Abstract

Background: Despite continued global and local initiatives to address nutritional problems, low- and middle-income countries are facing both malnutrition and non-communicable diseases, with about 80% of non-communicable disease-related deaths. There is a dearth of recent evidence on the extent and determinants of underweight, overweight, and obesity in this region, which is essential for guiding intervention programs. Thus, this study intended to provide insights into the current level of malnutrition among women of reproductive age in low- and middle-income countries.

Methods: A secondary analysis of Demographic and Health Survey data from 40 low- and middle-income countries was performed using a weighted sample of 1,044,340 women of reproductive age. Forest plots and line graph were used to present the pooled and regional estimates of underweight and overweight and obesity. A multilevel multinomial logistic regression models were fitted to identify determinants of malnutrition and models were compared based on the deviance and log-likelihood values. In the final model, statistical significance was determined using an adjusted relative risk ratio with the corresponding 95% confidence interval.

Results: The overall prevalence of obesity and overweight was 26.8% (95% CI: 26.7%-26.9%), with the highest rate in Jordan (67.2%) and lowest in Burundi (9.5%). Additionally, the level of underweight was found to be 15.6% [95% CI: 15.5%-15.7%], ranging from 1.3% in Jordan to 25.4% in Timor-Leste. Women of families with middle and rich wealth indexes, those aged 25-34 and 35-49, contraceptive users, those with primary and higher education, and urban

residents were more likely to be overweight or obese. In contrast, the results showed a lower likelihood of undernutrition among women in the households with middle [ARRR (95% CI): 0.80 (0.78-0.83)] and rich wealth indexes [ARRR (95% CI): 0.69 (0.67-0.71)], those with primary [ARRR (95% CI): 0.68 (0.66-0.71)] and higher education [ARRR (95% CI): 0.65 (0.63-0.67)], and women with media access [ARRR (95% CI): 0.79 (0.77-0.81)].

Conclusion: Over a quarter of women of reproductive age in LMICs were overweight or obese, with underweight affecting 15.6%. It was also found that the levels of overweight and obesity were higher than the rate for underweight, with wide variations across the countries. Thus, efforts to improve the modifiable risks can have a positive impact on reducing undernutrition and over-nutrition. Given the considerable variations between countries and survey periods, nutrition programs should also be tailored to the specific needs and cultural contexts of different regions within the study settings. Furthermore, the findings suggest the need for regular monitoring and evaluation of the existing nutritional interventions to assess their effectiveness.

Keywords: Low and middle-income countries, Underweight, Overweight, Obesity; Demographic and Health Survey, Determinants, Multilevel multinomial analysis, Women of reproductive age.

Background

Overweight and obesity are significant public health challenges in both developed and developing regions, disproportionately affecting women (40%) than men (35%) [1,2]. While overweight is marked by an excessive accumulation of body fat, obesity is a chronic and complex condition with substantial fat deposition. Both conditions pose varying degrees of

health risks associated with excessive body fat [1]. There are several linkages between obesity and health problems, including type 2 diabetes and heart disease, poor bone health, disturbed sleep, impaired reproductive function and mobility, and cancers of the reproductive organs [1,3,4].

Excessive weight gain in women is also associated with infertility, exacerbated polycystic ovary syndrome, heart disease, diabetes, and breast cancer due to changes in reproductive hormones [5,6]. Being overweight and obese during pregnancy is also linked with a higher risk of gestational diabetes and hypertension [7,8], and complications like postpartum hemorrhage, assisted delivery, and surgical site infections [9–11]. Furthermore, maternal exposure to these nutritional indices during the prenatal period elevates the risk of congenital malformations, preterm birth, perinatal death, and macrosomia [5,12,13].

Globally, about 4.0 million deaths and 120 million disability-adjusted life-years are attributed to overweight and obesity [14]. In 2022, approximately 1 in 8 individuals worldwide were living with obesity and 2.5 billion adults aged 18 years and older were overweight, with 890 million affected by obesity [1]. Additionally, 43% of women worldwide are affected by overweight or obesity [1].

The prevalence of overweight and obesity is on the rise in developing countries alongside economic development, reduced physical activity, and a shift towards high-energy and fatty diets [15,16]. For instance, among women, the level of overweight varies from 10% to 60% [17–25], while the prevalence of obesity ranges from 3% to 35% [17–20,23–26].

Previous studies showed that the factors associated with overweight and obesity include gender [8,27,28], age [8,27,29,30], marital status [26,27,29–32], wealth status [8,28–31], exposure to media [33,34], physical activities [28,34], alcohol consumption [31,34], snacking [31], education [29,30], occupation [30], contraceptive use [30], and place of residence [29,32].

Despite a continued effort of the International Fund for Agricultural Development and World Health Organization (WHO) to address the increasing rates of obesity and overweight, particularly in low- and middle-income countries (LMICs) [1,35], these regions are facing a double burden of malnutrition, where approximately 70% of the population are overweight or obese [35]. Additionally, these settings face an increased risk of non-communicable diseases (NCDs) such as diabetes and cardiovascular diseases, with about 80% of NCD-related deaths [35]. Despite these alarming figures, there is a lack of up-to-date evidence on the level and determinants of overweight and obesity in this region. Furthermore, previous studies in the region have utilized outdated DHS data, whereas our study aimed to bridge this gap by incorporating the most recent DHS datasets from the countries included. Thus, this study intended to provide insights into the current level of overweight and obesity among women of reproductive age (WRA) in LMICs using population survey data from 40 countries.

Methods

Data source and participants

Data were obtained from demographic and health surveys conducted between 2015 and 2022 in 40 LMICs (https://dhsprogram.com/data/dataset-admin/index.cfm). Before inclusion, the survey dataset of each country was checked for the availability of body mass index (BMI)

records, and countries with no data on this record were excluded from the analysis. After dropping records with missing observations and those with no unknown BMI data, a weighted sub-sample of 1,044,340 WRA (unweighted sample = 1,052,841) in all surveys was included in the analysis.

Variables and measurement

Dependent variable

The dependent variable was BMI, measured as the ratio of the weight in kilograms to the square of the height in meters (kg/m^2). Based on the WHO cut-off point, BMI was classified as underweight, normal, overweight, and obese if the BMI is < 18.5 kg/m^2, 18.5-24.9 kg/m^2, 25-29.9 $kg/m^{2,}$ and ≥ 30 kg/m^2, respectively [36]. For the analysis purpose, overweight and obesity were treated as one category.

Independent variables

Individual-level (level-1) variables included age, marital status, household wealth, health insurance coverage, women's and partner's education, employment, marital status, media exposure, household head, age at marriage, history of pregnancy loss, parity, number of under-five children, birth order, preceding birth interval, anemia status, contraceptive use, decision maker on women's health, source of drinking water, and toilet facility. While, region, place of residence, and distance to health facility were the community-level (level-2) variables.

Media exposure was constructed using the frequency of watching television, listening to the radio, and reading newspapers, which have three response options (i.e. not at all, less than once a week, and at least once a week). Using the DHS definition, women who indicated that

they watched television, listened to the radio, or read the newspaper at least once a week were classified as having media exposure, while those who did not meet this criterion were classified as not having exposure.

Data management and analysis

Data cleaning and analysis were conducted using Stata software version 17. Before analysis, the presence of the outcome variable (i.e. BMI) in the DHS dataset of each country was confirmed, and all study variables were assessed for missing data. Records with missing observations and unknown BMI were dropped from the analysis. Finally, the datasets of 40 LMICs were appended and weighted to compensate for the non-representativeness of the sample and obtain reliable estimates and standard errors.

To account for the hierarchical structure of DHS design and the polytomous nature of the outcome variable, a multilevel multinomial logistic regression model was applied to determine the effects of independent variables on underweight and overweight and obesity. Initially, bivariable multilevel logistic regression analysis was done and variables with a p-value of less than 0.25 in this analysis were considered for the multivariable regression model.

Fixed-effect multilevel analysis

Four hierarchical models were independently fitted to select the model that best fits the data. To test the random variability in the intercept, an empty model i.e. a model without an independent variable was fitted. In the second and third models, the effect of individual and community level variables on abnormal BMI was respectively assessed, while the combined

effect of both community and individual level variables on the outcome variable was simultaneously assessed in the fourth model.

In Model I (empty model), the likelihood of being underweight and overweight and obese is only a function of the clusters in which the women lived, which is indicated by a random intercept at the cluster level. In Model II (a model with level-1 variables), the probability of being underweight and overweight and obese was measured as a function of the cluster in which the women resided and individual-level characteristics. Model III (model with level-2 variables) assessed the probability of malnutrition as a function of cluster and community-level variables, while Model IV (model with both level-1 and level-2 variables) examined the combined effect of both level-1 and level-2 variables on the occurrence of underweight, overweight and obesity.

After fitting these models, models were compared based on log-likelihood (LL) and deviance (i.e. -2*LL) values to select the one that best fits the data. For instance, model IV has the lowest deviance and highest LL values and is hence considered the best-fit model for the final analysis. A generalized variance inflation factor [GVIF = $VIF^{[1/(2*df)]}$] was used to determine the presence of collinearity between explanatory variables and this value was less than five for all variables included in the final model, suggesting the non-existence of multi-collinearity. Finally, in the multivariable analysis, a p-value less than 0.05 and an adjusted relative risk ratio (ARRR) with the corresponding 95% confidence interval were used to identify determinants of underweight, overweight, and obesity.

Random-effect analysis (measures of variance)

Random variability in the level of underweight, overweight, and obesity across clusters was assessed by intra-class correlation coefficient (ICC), proportion change in variance (PCV), and median odds ratio (MOR). In this analysis, ICC indicates the total variance in the proportion of underweight, overweight, and obesity attributed to cluster differences. PCV (attributable percentage variability in the multilevel model) measured the differences in the prevalence of malnutrition attributed to community or individual-level characteristics. Heterogeneity in the risk of underweight and overweight and obesity among women in different clusters was assessed by MOR. It was measured as the median value of the odds of the outcome variable (i.e. abnormal BMI) between women living in the area at the highest risk of abnormal BMI compared to those in the clusters at the lowest risk of the event.

Ethical approval

Since we used secondary data, permission to access the data was granted from the Measure DHS official Database via an online request at http://www.dhsprogram.com. The survey procedures were also approved by the Institutional Review Board of the host country and ICF International. Additionally, written informed consent was initially obtained from the study participants during the collection of the survey data.

Results

Sociodemographic characteristics

Of 1,044,340 women of reproductive age, about three-fourths (75.2%) were from the Asia region, more than half (56.0%) resided in rural areas, and 433,278 (41.5%) were from rich households. Moreover, 357,587 (34.2) were between the ages of 15-24 years, less than one-

quarter (22.8%) had no formal schooling, and 733,430 (70.2%) were currently in a union (Table 1).

In this analysis, the highest prevalence of underweight was observed in women from Asia (17.0%), rural settings (17.4%), poor households (19.9%), and younger age groups (35.0%). Conversely, women from the African region (27.7%), urban dwelling (36.7%), rich households (35.7%), older age groups (37.6%), and those with higher education (31.0%) had higher rates of overweight and obesity than their respective categories (Table 1).

Table 1: Socio-economic characteristics of women of reproductive age in 40 low and middle-income countries, 2015 to 2022

Reproductive and health-related characteristics

More than half (58.3%) of women were married at 18 years of age or older, 136,858 (13.1%) had experienced pregnancy loss, and 479,261 (45.9%) were multiparous. Additionally, about half (51.9%) of WRA were anemic, 459,607 (44.0%) were using contraceptive methods, and nearly a quarter (26.7%) reported that distance to the nearest health facility was a big problem.

The highest proportion of undernutrition was observed among nulliparous women (24.7%), women with a birth order of 4 or more (19.8%), anemic women (18.1%), and those not using contraception (18.4%). Conversely, multiparous women (32.4%), those with a birth interval of at least 33 months (34.4%), and women using contraceptives (31.2%) had comparatively higher levels of overweight and obesity. Furthermore, the prevalence of overweight and obesity was higher among women with a history of pregnancy loss (37.2%), those with a lower birth order (31.1%), and those who made their own healthcare decisions (40%) (Table 2).

Table 2: Reproductive and health-related characteristics of women of reproductive age in 40 low and middle-income countries, 2015 to 2022

Prevalence of underweight

Our result showed that the overall prevalence of underweight among WRA in LMICs was 15.6% [95% CI: 15.5%-15.7%], with large variations across the included countries. The highest level of undernutrition was observed in Timor-Leste (25.4%), Ethiopia (21.1%), Chad (17.9%), and Burundi (17.6%), while Jordan (1.3%), Guatemala (2.9%), and Zambia (3.0%) had the lowest rates. Moreover, subgroup analysis revealed that Asia had the highest prevalence of underweight (17.0%), followed by Africa (11.2%) (Figure 1).

Figure 1: Pooled and national estimates of underweight among women of reproductive age in 40 low- and middle-income countries from 2015 to 2022.

Prevalence of overweight and obesity

The pooled prevalence of obesity and overweight among WRA in LMICs was 26.8% (95% IC: 26.7%-26.9%), of which overweight and obesity accounted for 18.8% [95% CI: 18.7%-18.9%] and 8.0% [95% CI: 7.9%-8.1], respectively. Higher rates were found in Jordan (67.2%), Turkey (62.2%), and Zambia (61.6%), while the lowest prevalence was noted in Burundi (9.5%) and Timor-Leste (9.9%). Additionally, the analysis noted the highest prevalence of overnutrition among women in Latin America, Caribbean, and Europe (42.7%), followed by Africa (40.6%) (Figure 2).

Figure 2: Pooled and national estimates of overweight and obesity among women of reproductive age in 40 low- and middle-income countries from 2015 to 2022.

Of forty countries included in the analysis, the rate of overweight and obesity exceeded the prevalence of underweight in 35 countries. The exceptions were five countries (Burundi, Ethiopia, Madagascar, Chad, and Timor-Leste) where the highest level of undernutrition was observed (Table 3).

Table 3: Country-specific prevalence of underweight and overweight and obesity among women of reproductive age in 40 low- and middle-income countries, 2015 to 2022

Change in the level of underweight and overweight and obesity over survey years

Figure 3 shows the change in the level of underweight and overweight and obesity across the survey years in 40 LMICs. This result indicated that the level of obesity and overweight surpassed that of underweight during all survey periods, with the peak prevalence in 2017 (38.5%) and 2019 (38.3%). Our analysis also showed a decline in undernutrition from 18.1% to 9.4% between 2020/21 and 2022, while the level of overweight and obesity rose by about 10%, from 23.8% to 33.9% during the same period. Overall, both undernutrition and overnutrition were slightly increased between 2015 and 2022 (Figure 3).

Figure 3: Change in the level of underweight and overweight and obesity among women of reproductive age in 40 low- and middle-income countries from 2015 to 2022.

Random effect result (measures of variations)

A random effect analysis result indicated that 44% of the variability in the level of malnutrition was due to differences at the cluster level (as indicated by the ICC value of model 1), while 31% of the total variability was explained by both individual and community-level factors (ICC value of model IV). In the final model (model IV), the PCV value also shows that 29% of the variation

in malnutrition among WRA was explained by the effect of both individual and community-level covariates. Furthermore, the MOR of an empty model indicated the existence of heterogeneity in the magnitude of underweight and overweight and obesity across clusters. This implies that WRA in the clusters with higher levels of malnutrition were 1.72 times more likely to suffer from malnutrition than those in the clusters with lower prevalence (Table 4).

Table 4: Measures of variations and model statistics summary for determinants of underweight and overweight and obesity among women of reproductive age in 40 LMICs, 2015 to 2022

Determinants of underweight and overweight and obesity

The result showed that the risk of overweight and obesity was higher for WRA of households with middle [ARRR (95% CI): 1.31 (1.29-1.34)] and rich wealth indexes [ARRR (95% CI): 1.64 (1.61-1.67)] and those aged 25-34 [ARRR (95% CI): 2.11 (2.05-2.17)] and 35-49 years [ARRR (95% CI): 3.64 (3.53-3.74)]. Moreover, contraceptive use [1.22 (1.20-1.25)], attending primary [ARRR (95% CI): 1.56 (1.52-1.59)] and higher education [ARRR (95% CI): 1.91 (1.87-1.96)], and living in urban areas [ARRR (95% CI): 1.73 (1.70-1.76)] were associated with an increased risk overnutrition.

Our result also revealed that the likelihood of undernutrition was lower for women in the households with middle [ARRR (95% CI): 0.80 (0.78-0.83)] and rich wealth indexes [ARRR (95% CI): 0.69 (0.67-0.71)], those with primary [ARRR (95% CI): 0.68 (0.66-0.71)] and higher education [ARRR (95% CI): 0.65 (0.63-0.67)], and women with media access [ARRR (95% CI): 0.79 (0.77-0.81)]. Additionally, age, employment status, media exposure, women's involvement in

healthcare decisions, parity, contraceptive use, and place of residence were significantly associated with undernutrition (Table 5).

Table 5: Determinants of underweight and overweight and obesity among women of reproductive age in 40 low and middle-income countries, 2015 to 2022

Discussion

This study assessed the level and determinants of underweight, overweight, and obesity among WRA in 40 LMICs. The pooled prevalence of overweight and obesity was 26.8% (95% CI: 26.7%-26.9%). The current result is in line with prior studies in England (26%) [42] and Mali (26.9%) [41], but higher than the prevalence reported from the studies done in Ethiopia (15%) [24], Malawi (23%) [37], Sub-Saharan Africa (22.6%) [38,39], and Uganda (22.4%) [18]. Contrarily, our finding is lower compared to the studies conducted in South Africa (27%) [41], Nigeria (30%) [42], Cameroon (50%) [43], Egypt (85%) [44], Kenya (30%) [30], Zimbabwe (46.5%) [45], and Australia (60%) [46]. These disparities could be attributed to divergent levels of economic development, urbanization promoting sedentary lifestyles, culture-specific food taboos and dietary practices, and variations in levels of physical activity and nutritional awareness across the study settings.

Our findings also revealed that the prevalence of underweight among WRA in LMICs was 15.6% [95% CI: 15.5%-15.7%], with substantial variations among the countries studied. This finding is lower than the rates reported in India (20%) [47], South Asia (24%) [48], and LMICs (42%) [48]. However, our finding is higher than that reported in the studies done in Bangladesh (12%) [49], Sierra Leone (6.7%) [50], global prevalence (9.7%) [51], Myanmar (14%) [52], Indonesia (14%)

[53], and Botswana (10%) [54]. These variations can be attributed to differences in socioeconomic status, disparities in education and health awareness, cultural dietary practices, regional food security discrepancies, and access to nutritional education across different settings.

Similar to the results of the studies done in Kenya [55], Bangladesh [56], India [57], Nepal [56], and SSA [58], our analysis revealed that women from households with middle and rich wealth indexes exhibited a higher risk of overweight and obesity compared to their counterparts. Our study also found that belonging to wealthy families is associated with lower odds of being underweight compared to women of families with poor wealth index. This aligns with previous studies in various contexts [59–61] showing similar associations. This could be because women of households with middle and rich wealth indexes have relatively easier access to calorie-dense diets, which can lead to increased calorie intake. Additionally, their higher socioeconomic status tends to correlate with lower physical activity levels and increased sedentary behavior. Furthermore, psychosocial factors like stress associated with higher socioeconomic status can impact eating behaviors, further contributing to overweight and obesity [62].

In this analysis, women aged between 25-34 and 35-49 years were more likely to be overweight and obese than younger women. This aligns with the findings from the previous studies in different settings [63–66]. This might be due to biological changes and shifts in lifestyle as women age increases. Factors such as metabolic changes, reduced physical activity, and altered dietary habits might likely contributed to the observed result. Our finding also showed a lower risk of underweight, overweight and obesity among women who married at the age of 18 or older. The possible justification could be that delaying marriage allows women more time to

develop healthier lifestyle habits, such as improved diet and regular exercise. Additionally, it correlates with higher education and socio-economic status, factors known to contribute to better health outcomes and reduced risk of weight-related issues [67].

Our study found a higher risk of overweight and obesity among women with primary and higher education, alongside lower likelihood of being underweight in these groups. These results align with previous studies across different settings [68–70]. The possible reason might be that higher educational attainment may contribute to better weight management practices by providing increased access to resources and knowledge about nutrition and healthy lifestyles. Furthermore, higher educational attainment often leads to greater economic stability, which can improve access to healthier food options. This improved access may contribute to a lower risk of underweight among women with higher education levels. However, on the other side, it can increase the risk of overweight and obesity due to potential changes in dietary habits and sedentary lifestyles (always in cars, with maid in houses, low levels of physical activity) associated with socio-economic status like [71].

Access to mass media was also identified as a significant factor linked with both undernutrition and overnutrition. The study found that media access was associated with a higher risk of obesity and overweight and a lower risk of underweight. Similarly, previous studies in both developed and developing nations [72–75] have reported comparable associations. Potential explanations include the promotion of unhealthy foods and sedentary behaviors via media, which can contribute to weight gain and obesity. Unrealistic body ideals portrayed in the media may also lead to body image issues and disordered eating, impacting both obesity and underweight trends. However, access to media also creates an opportunity for health literacy

on dietary practices, potentially reducing the risks of malnutrition, and influencing cultural norms regarding dietary habits and weight management practices [76].

We found that employed women had a lower likelihood of undernutrition and overnutrition compared to their non-employed counterparts. Studies conducted in Malaysia, Ethiopia, and Mexico have reported similar findings [77–79]. The possible explanation could be that employed women experience higher stress levels and time constraints, leading to irregular eating habits and reliance on convenient but less nutritious food choices compared to their counterparts.

Similar to studies in both high- and low-income countries [23,80–82], our analysis showed that multiparous women have a higher risk of overweight and obesity than primipara and nullipara women. This could be due to the cumulative effect of weight gain across successive pregnancies, where weight gained during each pregnancy is not completely lost before the next pregnancy. Additionally, the demands of childcare and family responsibilities may reduce opportunities for physical activity and healthy meal planning, further contributing to the higher likelihood of overweight and obesity among multipara [80].

Contraceptive use was also found to be associated with overweight and obesity. The study showed that women using contraceptives are more likely to be overweight or obese compared to non-users. The current findings align with previous studies [83–85]. This can be explained by the fact that hormonal contraceptives alter hormone levels, potentially influencing metabolism, fat deposition, and appetite regulation, which promote weight gain. Additionally, reduced concern about pregnancy-related weight changes among contraceptive users may lead to

decreased attention to diet and exercise, further contributing to a higher risk of overweight and obesity [86,87].

Furthermore, our analysis revealed that women whose healthcare relied solely on their husbands' decisions or who made joint decisions with their partners were more likely to be underweight compared to other women. Consistently, previous studies have shown similar associations [88–90]. This might be because societal norms and gender roles limit women's autonomy in practicing healthy behaviors and access to nutritious food and healthcare. These constraints, compounded by cultural expectations and lower social support within decision-making frameworks, ultimately contribute to inadequate nutrition and healthcare utilization, leading to poorer health outcomes such as underweight.

In addition, it was found that compared to African women, women in Asia, Latin America, the Caribbean, and Europe had a higher risk of being overweight and obese. Additionally, women in Asia also faced a double burden of malnutrition, with a significant risk of underweight and overweight and obesity. Likewise, previous studies in LMICs have shown similar trends in overweight and obesity [91–93]. The possible reason could be that in rapidly urbanizing and economically growing regions like Asia, Latin America, the Caribbean, and Europe, increased sedentary behavior, higher consumption of processed foods, and reduced physical activity contribute to rising obesity rates. Conversely, in less developed settings like Asia and Africa, traditional diets and more active lifestyles may help maintain a lower risk of obesity [94].

Urban residence was also significantly associated with an increased risk of overweight and obesity. Women residing in urban areas exhibited a higher risk of overweight and obesity

compared to their rural counterparts. This is consistent with the previous studies in SSA [58], Nigeria [95], Myanmar [96], and Ethiopia [97]. The possible explanation could be that in rural areas, women's active engagement in agriculture helps limit weight gain compared to urban women. Additionally, rural lifestyles generally involve less exposure to sedentary behavior, modern transportation, reduced physical activity, and consumption of high-fat, energy-dense foods typical of urban settings [1].

Strengths and limitations

The strengths of this analysis lie in the use of recent population survey data from 40 countries, and the application of advanced statistical methods i.e. multilevel multinomial modeling to account for the nested structure of the DHS data and the polytomous nature of the outcome variable. However, it is important to acknowledge certain limitations when interpreting the study's findings. These include potential over or underestimation of estimates due to a large number of missing observations (274,180 missing), and differences in the survey year, number of countries and sample sizes across the regions included in the analysis might affect the regional estimates. The use of BMI for assessing nutritional status does not differentiate between excess fat and bone mass and does not provide information about the distribution of body fat. Additionally, the study did not examine the effect of physical activity, dietary patterns, and household food security status on the outcome variable since DHS does not gather data on these variables.

Conclusion

The pooled prevalence of obesity and overweight among WRA in LMICs was 26.8%, while underweight affected 15.6%. Our analysis also revealed that the levels of overweight and obesity were higher than the rate for underweight, with wide variations across the countries and survey periods. Moreover, the result showed that factors like household wealth, maternal education, age, place of residence, contraceptive use, media access, healthcare decisions, and employment status bidirectionally influenced both underweight and overweight and obesity. Efforts to improve household wealth, maternal literacy and healthcare, and employment opportunities for women can have a positive impact on reducing both underweight and overweight and obesity. Given the considerable variations between countries and survey time, nutrition programs should be tailored to the specific needs and cultural contexts of different regions within the study settings. Furthermore, the study's findings suggest the need for regular monitoring and evaluation of the existing nutritional interventions to assess their effectiveness.

Abbreviations

ARRR: Adjusted Relative Risk Ratio; AIC: BMI: Body Mass Index; DHS: Demographic and Health Survey; GVIF: Generalized Variance Inflation Factor; ICC: Intra-class correlation; LL: Log-likelihood; LMICs: Lower- and Middle-Income Countries; MOR: Median Odds Ratio; NCD: Non-communicable Diseases; PCV: Proportion Change in Variance; SSA: Sub-Saharan Africa; WHO: World Health Organization; WRA: Women of Reproductive Age.

Acknowledgments

The authors thank ICF International for granting access to the dataset used in this study.

References

1. Obesity and overweight [Internet]. [cited 2024 Jun 18]. Available from: https://www.who.int/news-room/fact-sheets/detail/obesity-and-overweight

2. Chong B, Jayabaskaran J, Kong G, Chan YH, Chin YH, Goh R, et al. Trends and predictions of malnutrition and obesity in 204 countries and territories: an analysis of the Global Burden of Disease Study 2019. eClinicalMedicine [Internet]. 2023 Mar 1 [cited 2024 Jun 18];57. Available from: https://www.thelancet.com/journals/eclinm/article/PIIS2589-5370(23)00027-5/fulltext

3. Fruh SM. Obesity: Risk factors, complications, and strategies for sustainable long-term weight management. J Am Assoc Nurse Pract. 2017 Oct;29(S1):S3.

4. Cefalu WT, Bray GA, Home PD, Garvey WT, Klein S, Pi-Sunyer FX, et al. Advances in the Science, Treatment, and Prevention of the Disease of Obesity: Reflections From a Diabetes Care Editors' Expert Forum. Diabetes Care. 2015 Jul 14;38(8):1567–82.

5. Overweight and Obesity - Obesity and Women's Health | NHLBI, NIH [Internet]. 2022 [cited 2024 Jun 18]. Available from: https://www.nhlbi.nih.gov/health/overweight-and-obesity/women

6. Obesity: Health consequences of being overweight [Internet]. [cited 2024 Jun 18]. Available from: https://www.who.int/news-room/questions-and-answers/item/obesity-health-consequences-of-being-overweight

7. Hossain MB, Khan JR, Gupta RD. Role of hypertension in the association of overweight and obesity with diabetes among adults in Bangladesh: a population-based, cross-sectional nationally representative survey. BMJ Open. 2021 Jul 1;11(7):e050493.

8. Prevalence and factors associated with overweight and obesity among adults in Hawassa city, southern Ethiopia: a community based cross-sectional study | BMC Obesity | Full Text

[Internet]. [cited 2024 Jun 18]. Available from: https://bmcobes.biomedcentral.com/articles/10.1186/s40608-019-0227-7

9. Martins I, Ayres-de-Campos D. 41 - Assisted vaginal delivery in obese women. In: Mahmood T, Lim CC, editors. Handbook of Obesity in Obstetrics and Gynecology [Internet]. Academic Press; 2023 [cited 2024 Jun 21]. p. 353–6. Available from: https://www.sciencedirect.com/science/article/pii/B9780323899048000202

10. Butwick AJ, Abreo A, Bateman BT, Lee HC, El-Sayed YY, Stephansson O, et al. The Effect of Maternal Body Mass Index on Postpartum Hemorrhage. Anesthesiology. 2018 Apr;128(4):774–83.

11. Mitchell CJ, Adkins L, Tucker A, Brown H, Siegel A, Dotter-Katz S. Impact of Excess Weight Gain on Risk of Postpartum Infection in Class III Obesity. AJP Rep. 2020 Jul;10(3):e213–6.

12. Being overweight in pregnancy and after birth [Internet]. RCOG. [cited 2024 Jun 18]. Available from: https://www.rcog.org.uk/for-the-public/browse-our-patient-information/being-overweight-in-pregnancy-and-after-birth/

13. Obesity and Pregnancy | ACOG [Internet]. [cited 2024 Jun 18]. Available from: https://www.acog.org/womens-health/faqs/obesity-and-pregnancy

14. null null. Health Effects of Overweight and Obesity in 195 Countries over 25 Years. N Engl J Med. 2017;377(1):13–27.

15. Obesity [Internet]. [cited 2024 Jun 18]. Available from: https://www.who.int/health-topics/obesity

16. Weight of nations: a socioeconomic analysis of women in low- to middle-income countries - ScienceDirect [Internet]. [cited 2024 Jun 18]. Available from: https://www.sciencedirect.com/science/article/pii/S0002916523020658?via%3Dihub

17. Araújo FG, Velasquez-Melendez G, Felisbino-Mendes MS. Prevalence trends of overweight, obesity, diabetes and hypertension among Brazilian women of reproductive age based on sociodemographic characteristics. Health Care Women Int. 2019 Apr 3;40(4):386–406.

18. Yaya S, Ghose B. Trend in overweight and obesity among women of reproductive age in Uganda: 1995–2016. Obes Sci Pract. 2019 Jul 11;5(4):312–23.

19. Tagbo SO, Abebe D, Oguoma VM. Overweight and obesity among non-pregnant women of reproductive age in Nigeria: findings from the 2008–2018 Nigerian Demographic and Health Survey. Public Health. 2021 Sep 1;198:348–57.

20. Um S, Yom A, Muir JA, Sopheab H. Overweight and obesity among women at reproductive age in Cambodia: Data analysis of Cambodia Demographic and Health Survey 2014. PLOS Glob Public Health. 2023 Mar 31;3(3):e0001768.

21. Mosha D, Paulo HA, Mwanyika-Sando M, Mboya IB, Madzorera I, Leyna GH, et al. Risk factors for overweight and obesity among women of reproductive age in Dar es Salaam, Tanzania. BMC Nutr. 2021 Jul 16;7(1):37.

22. Mukora-Mutseyekwa F, Zeeb H, Nengomasha L, Kofi Adjei N. Trends in Prevalence and Related Risk Factors of Overweight and Obesity among Women of Reproductive Age in Zimbabwe, 2005–2015. Int J Environ Res Public Health. 2019 Jan;16(15):2758.

23. Nglazi MD, Ataguba JEO. Overweight and obesity in non-pregnant women of childbearing age in South Africa: subgroup regression analyses of survey data from 1998 to 2017. BMC Public Health. 2022 Feb 25;22(1):395.

24. Mengesha Kassie A, Beletew Abate B, Wudu Kassaw M. Education and prevalence of overweight and obesity among reproductive age group women in Ethiopia: analysis of the 2016 Ethiopian demographic and health survey data. BMC Public Health. 2020 Jul 31;20(1):1189.

25. Kordas K, Centeno ZYF, Pachón H, Soto AZJ. Being Overweight or Obese Is Associated with Lower Prevalence of Anemia among Colombian Women of Reproductive Age. J Nutr. 2013 Feb 1;143(2):175–81.

26. Balarajan Y, Villamor E. Nationally Representative Surveys Show Recent Increases in the Prevalence of Overweight and Obesity among Women of Reproductive Age in Bangladesh, Nepal, and India, ,. J Nutr. 2009 Nov 1;139(11):2139–44.

27. Ajayi IO, Adebamowo C, Adami HO, Dalal S, Diamond MB, Bajunirwe F, et al. Urban–rural and geographic differences in overweight and obesity in four sub-Saharan African adult populations: a multi-country cross-sectional study. BMC Public Health. 2016 Oct 28;16(1):1126.

28. Shayo GA, Mugusi FM. Prevalence of obesity and associated risk factors among adults in Kinondoni municipal district, Dar es Salaam Tanzania. BMC Public Health. 2011 May 23;11(1):365.

29. Delbiso TD, Rodriguez-Llanes JM, Altare C, Masquelier B, Guha-Sapir D. Health at the borders: Bayesian multilevel analysis of women's malnutrition determinants in Ethiopia. Glob Health Action. 2016 Jul 4;9:10.3402/gha.v9.30204.

30. Mkuu RS. Prevalence and Predictors of Overweight and Obesity Among Kenyan Women. Prev Chronic Dis [Internet]. 2018 [cited 2024 Jun 18];15. Available from: https://www.cdc.gov/pcd/issues/2018/17_0401.htm

31. Dagne S, Gelaw YA, Abebe Z, Wassie MM. <p>Factors associated with overweight and obesity among adults in northeast Ethiopia: a cross-sectional study</p>. Diabetes Metab Syndr Obes. 2019 Mar 22;12:391–9.

32. Janghorbani M, Amini M, Willett WC, Gouya MM, Delavari A, Alikhani S, et al. First Nationwide Survey of Prevalence of Overweight, Underweight, and Abdominal Obesity in Iranian Adults. Obesity. 2007;15(11):2797–808.

33. Musaiger A, Al-Mannai M. Association between exposure to media and body weight concern among female university students in five Arab countries: A preliminary cross-cultural study. J Biosoc Sci. 2013 Jun 12;46:1–8.

34. Darebo T, Mesfin A, Gebremedhin S. Prevalence and factors associated with overweight and obesity among adults in Hawassa city, southern Ethiopia: a community based cross-sectional study. BMC Obes. 2019 Mar 4;6:8.

35. We must do more to curb obesity and overweight in low- and middle-income countries [Internet]. IFAD. [cited 2024 Jun 18]. Available from: https://www.ifad.org/en/web/latest/-/we-must-do-more-to-curb-obesity-and-overweight-in-low-and-middle-income-countries

36. Malnutrition in women [Internet]. [cited 2024 Nov 22]. Available from: https://www.who.int/data/nutrition/nlis/info/malnutrition-in-women

37. Mndala L, Kudale A. Distribution and social determinants of overweight and obesity: a cross-sectional study of non-pregnant adult women from the Malawi Demographic and Health Survey (2015-2016). Epidemiol Health. 2019;41:e2019039.

38. Double burden of malnutrition and associated factors among women of reproductive age in sub-Saharan Africa: a multilevel multinomial logistic regression analysis | BMJ Open [Internet]. [cited 2024 Dec 20]. Available from: https://bmjopen.bmj.com/content/14/2/e073447.long

39. Neupane S, K. C. P, Doku DT. Overweight and obesity among women: analysis of demographic and health survey data from 32 Sub-Saharan African Countries. BMC Public Health. 2015 Dec;16(1):1–9.

40. Double burden of malnutrition and associated factors among women of reproductive age in sub-Saharan Africa: a multilevel multinomial logistic regression analysis | BMJ Open [Internet]. [cited 2024 Dec 20]. Available from: https://bmjopen.bmj.com/content/14/2/e073447.long

41. Sartorius B, Veerman LJ, Manyema M, Chola L, Hofman K. Determinants of Obesity and Associated Population Attributability, South Africa: Empirical Evidence from a National Panel Survey, 2008-2012. PLoS ONE. 2015 Jun 10;10(6):e0130218.

42. Chukwuonye II, John, Ohagwu, Ejiji, Imoh, Chuku, et al. Prevalence of overweight and obesity in adult Nigerians – a systematic review. Diabetes Metab Syndr Obes Targets Ther. 2013 Jan;43.

43. Simo LP, Agbor VN, Temgoua FZ, Fozeu LCF, Bonghaseh DT, Mbonda AGN, et al. Prevalence and factors associated with overweight and obesity in selected health areas in a rural health district in Cameroon: a cross-sectional analysis. BMC Public Health. 2021 Mar 10;21(1):475.

44. Reyes Matos U, Mesenburg MA, Victora CG. Socioeconomic inequalities in the prevalence of underweight, overweight, and obesity among women aged 20–49 in low- and middle-income countries. Int J Obes. 2020 Mar;44(3):609–16.

45. Mangemba NT, San Sebastian M. Societal risk factors for overweight and obesity in women in Zimbabwe: a cross-sectional study. BMC Public Health. 2020 Jan 28;20(1):103.

46. Overweight and obesity, Summary [Internet]. Australian Institute of Health and Welfare. 2024 [cited 2024 Jun 22]. Available from: https://www.aihw.gov.au/reports/overweight-obesity/overweight-and-obesity/contents/overweight-and-obesity

47. Pengpid S, Peltzer K. Prevalence and correlates of underweight and overweight/obesity among women in India: results from the National Family Health Survey 2015–2016. Diabetes Metab Syndr Obes Targets Ther. 2019 May;Volume 12:647–53.

48. Victora CG, Christian P, Vidaletti LP, Gatica-Domínguez G, Menon P, Black RE. Revisiting maternal and child undernutrition in low-income and middle-income countries: variable progress towards an unfinished agenda. Lancet Lond Engl. 2021 Apr 10;397(10282):1388–99.

49. Hasan MdM, Ahmed S, Soares Magalhaes RJ, Fatima Y, Biswas T, Mamun AA. Double Burden of Malnutrition Among Women of Reproductive Age in Low- and Middle-Income Countries: Progress Achieved and Opportunities for Meeting the Global Target. Curr Dev Nutr. 2020 Jun 1;4:nzaa053_044.

50. Ikoona EN, Toure MA, Njenga A, Namulemo L, Kaluya R, Kamara K, et al. Prevalence and factors associated with underweight among 15–49-year-old women in Sierra Leone: a secondary data analysis of Sierra Leone demographic health survey of 2019. BMC Womens Health. 2023 Apr 22;23(1):192.

51. Trends in adult body-mass index in 200 countries from 1975 to 2014: a pooled analysis of 1698 population-based measurement studies with 19·2 million participants. The Lancet. 2016 Apr 2;387(10026):1377–96.

52. Hong SA, Peltzer K, Lwin KT, Aung LS. The prevalence of underweight, overweight and obesity and their related socio-demographic and lifestyle factors among adult women in Myanmar, 2015-16. PLOS ONE. 2018 Mar 16;13(3):e0194454.

53. Hanandita W, Tampubolon G. The double burden of malnutrition in Indonesia: Social determinants and geographical variations. SSM - Popul Health. 2015 Dec 1;1:16–25.

54. Letamo G, Navaneetham K. Prevalence and Determinants of Adult Under-Nutrition in Botswana. PLOS ONE. 2014 Jul 23;9(7):e102675.

55. Haregu TN, Mohamed SF, Muthuri S, Khayeka-Wandabwa C, Kyobutungi C. Body mass index and wealth index: positively correlated indicators of health and wealth inequalities in Nairobi slums. Glob Health Epidemiol Genomics. 2018 Jun 4;3:e11.

56. Bishwajit G. Household wealth status and overweight and obesity among adult women in Bangladesh and Nepal. Obes Sci Pract. 2017;3(2):185–92.

57. Let S, Tiwari S, Singh A, Chakrabarty M. Spatiotemporal change in wealth-based inequalities in overweight/obesity among women of reproductive age in India, 2015–2021. Clin Epidemiol Glob Health. 2023 Nov 1;24:101458.

58. Atsu P, Mohammed A, Adu C, Aboagye RG, Ahinkorah BO, Seidu AA. Residence-based inequalities in overweight/obesity in sub-Saharan Africa: a multivariate non-linear decomposition analysis. Trop Med Health. 2024 Apr 7;52(1):29.

59. Rahman A, Sathi NJ. Sociodemographic Risk Factors of Being Underweight Among Ever-Married Bangladeshi Women of Reproductive Age: A Multilevel Analysis. Asia Pac J Public Health [Internet]. 2020 Dec 8 [cited 2024 Jun 23]; Available from: https://journals.sagepub.com/doi/10.1177/1010539520979924

60. Wealth index and other behavioral and sociodemographic characteristics associated with body mass index in Ethiopia - PMC [Internet]. [cited 2024 Jun 23]. Available from: https://www.ncbi.nlm.nih.gov/pmc/articles/PMC8142017/

61. Heo J, Yu SY, Yi J, Nam YS, Son DT, Oh J, et al. Wealth gradient-based divergence in the prevalence of underweight among women by marital status in Quoc Oai district, Vietnam. Glob Health Action. 2018 Jan;11(1):1449430.

62. Anekwe CV, Jarrell AR, Townsend MJ, Gaudier GI, Hiserodt JM, Cody Stanford F. Socioeconomics of Obesity. Curr Obes Rep. 2020 Sep;9(3):272–9.

63. Yussif MT, Morrison AE, Annan RA. 10-year level, trends and socio-demographic disparities of obesity among Ghanaian adults—A systematic review and meta-analysis of observational studies. PLOS Glob Public Health. 2024 Jan 25;4(1):e0002844.

64. Adult body weight trends in 27 urban populations of Brazil from 2006 to 2016: A population-based study - PubMed [Internet]. [cited 2024 Jun 24]. Available from: https://pubmed.ncbi.nlm.nih.gov/30840675/

65. Rathnayake N, Rathnayake H, Lekamwasam S. Age-Related Trends in Body Composition among Women Aged 20–80 Years: A Cross-Sectional Study. J Obes. 2022 Feb 2;2022:4767793.

66. Lima AP de, Nunes AP de OB, Nicoletti CF, Benatti FB. Trend in the Prevalence of Overweight and Obese Adults in São Paulo, Brazil: Analysis between the Years 2006 and 2019. Int J Environ Res Public Health. 2024 Apr;21(4):502.

67. Cobb LK, McAdams-DeMarco MA, Gudzune KA, Anderson CAM, Demerath E, Woodward M, et al. Changes in Body Mass Index and Obesity Risk in Married Couples Over 25 Years. Am J Epidemiol. 2016 Mar 1;183(5):435–43.

68. Assari S. Education Attainment and Obesity : Differential Returns Based on Sexual Orientation. Behav Sci Basel Switz. 2019 Jan 29;9(2):16.

69. Education and obesity at age 40 among American adults - PubMed [Internet]. [cited 2024 Jun 24]. Available from: https://pubmed.ncbi.nlm.nih.gov/23246398/

70. Sart G, Bayar Y, Danilina M. Impact of educational attainment and economic globalization on obesity in adult females and males: Empirical evidence from BRICS economies. Front Public Health [Internet]. 2023 Feb 14 [cited 2024 Jun 24];11. Available from: https://www.frontiersin.org/journals/public-health/articles/10.3389/fpubh.2023.1102359/full

71. Ethnicity & Disease | JSTOR [Internet]. [cited 2024 Jun 24]. Available from: https://www.jstor.org/journal/ethnicitydisease

72. Shiyab W, Halcomb E, Rolls K, Ferguson C. The Impact of Social Media Interventions on Weight Reduction and Physical Activity Improvement Among Healthy Adults: Systematic Review. J Med Internet Res. 2023 Mar 16;25:e38429.

73. Internet Use in Relation to Overweight and Obesity: A Systematic Review and Meta-Analysis of Cross-Sectional Studies - PMC [Internet]. [cited 2024 Jun 24]. Available from: https://www.ncbi.nlm.nih.gov/pmc/articles/PMC7442323/

74. Liu Q, Li X. The Interactions of Media Use, Obesity, and Suboptimal Health Status: A Nationwide Time-Trend Study in China. Int J Environ Res Public Health. 2021 Dec 15;18(24):13214.

75. Loh YL, Yaw QP, Lau Y. Social media-based interventions for adults with obesity and overweight: a meta-analysis and meta-regression. Int J Obes. 2023 Jul;47(7):606–21.

76. Stanford FC, Tauqeer Z, Kyle TK. Media and Its Influence on Obesity. Curr Obes Rep. 2018 Jun;7(2):186–92.

77. Full article: Obesity and employment as predictors of diabetes in Mexican Americans: findings from a longitudinal study [Internet]. [cited 2024 Jun 24]. Available from: https://www.tandfonline.com/doi/full/10.1080/00036840701335629

78. Mesfin BA, Argaw AT, Getahun Negash F, Emiru DA, Aserese AD, Matebe GY. Minimum Dietary Diversity and Associated Factors Among Pregnant Women Living in Arba Minch Health and Demographic Surveillance Sites, Southern Ethiopia, 2022. Health Serv Res Manag Epidemiol. 2023 Jan 1;10:23333928231166671.

79. Hamid SBA, Razali MFM, Suhaimi FA, Nor NM. Association of Food Insecurity and Weight Status of Women in Tuba Island, Malaysia. Curr Res Nutr Food Sci J. 2021 Aug 27;9(2):441–9.

80. TAGHDIR M, ALIMOHAMADI Y, SEPANDI M, REZAIANZADEH A, ABBASZADEH S, MAHMUD FM. Association between parity and obesity: a cross sectional study on 6,447 Iranian females. J Prev Med Hyg. 2020 Oct 6;61(3):E476–81.

81. Sun MH, Wen ZY, Wang R, Gao C, Yin JL, Chang YJ, et al. Parity and Metabolic Syndrome Risk: A Systematic Review and Meta-Analysis of 15 Observational Studies With 62,095 Women. Front Med. 2022 Jul 12;9:926944.

82. Abrams B, Heggeseth B, Rehkopf D, Davis E. Parity and body mass index in US women: A prospective 25-year study. Obesity. 2013;21(8):1514–8.

83. Endalifer ML, Diress Alen G, Addisu A, Linger B. The association between combined oral contraceptive use and overweight/obesity: a secondary data analysis of the 2016 Ethiopia Demographic and Health Survey. BMJ Open. 2020 Dec;10(12):e039229.

84. Simmons KB, Edelman AB. Hormonal contraception and obesity. Fertil Steril. 2016 Nov 1;106(6):1282–8.

85. Lopez LM, Bernholc A, Chen M, Grey TW, Otterness C, Westhoff C, et al. Hormonal contraceptives for contraception in overweight or obese women - Lopez, LM - 2016 | Cochrane Library. [cited 2024 Jun 24]; Available from: https://www.cochranelibrary.com/cdsr/doi/10.1002/14651858.CD008452.pub4/full

86. Progestin-only contraceptives: effects on weight - Lopez, LM - 2016 | Cochrane Library [Internet]. [cited 2024 Jun 24]. Available from: https://www.cochranelibrary.com/cdsr/doi/10.1002/14651858.CD008815.pub4/references

87. Meta-Analysis the Effect of Hormonal Contraception on the Weight Gain and Hypertension in Women of Reproductive Age | Journal of Maternal and Child Health [Internet]. [cited 2024 Jun 24]. Available from: https://thejmch.com/index.php/thejmch/article/view/868

88. Kedir H, Berhane Y, Worku A. Magnitude and determinants of malnutrition among pregnant women in eastern Ethiopia: evidence from rural, community-based setting. Matern Child Nutr. 2016 Jan;12(1):51–63.

89. Maitra C, Sethi V, Unisa S, Shankar S. Household Food Insecurity and Maternal and Child Nutritional Status: Evidence from Maharashtra. Rev Income Wealth. 2019;65(S1):S63–101.

90. Yamada M, Hapsari ED, Matsuo H. Behaviors toward Noncommunicable Diseases Prevention and their Relationship with Physical Health Status among Community-dwelling, Middle-aged and Older women in Indonesia. Int J Environ Res Public Health. 2020 Mar 30;17(7):2332.

91. Boutari C, Mantzoros CS. A 2022 update on the epidemiology of obesity and a call to action: as its twin COVID-19 pandemic appears to be receding, the obesity and dysmetabolism pandemic continues to rage on. Metabolism. 2022 Aug;133:155217.

92. New UN report: 43.2 million people suffer from hunger in Latin America and the Caribbean, and the region has higher levels of overweight and obesity than the global estimate [Internet]. FAO-RLC. [cited 2024 Jun 25]. Available from: https://www.fao.org/americas/news/news-detail/New-UN-report-43-2-million-people-suffer-from-hunger-in-Latin-America-and-the-Caribbean-and-the-region-has-higher-levels-of-overweight-and-obesity-than-the-global-estimate-/en

93. Obesity and overweight populations in Latin America [Internet]. [cited 2024 Jun 25]. Available from: https://www.thelancet.com/campaigns/kidney/updates/obesity-and-overweight-populations-in-latin-america

94. Lm J, Mm S, Bm P. Recent trends in the prevalence of under- and overweight among adolescent girls in low- and middle-income countries. Pediatr Obes [Internet]. 2015 Dec [cited 2024 Jun 25];10(6). Available from: https://pubmed.ncbi.nlm.nih.gov/25558987/

95. Baruwa OJ, Gbadebo BM, Adeleye OJ, Tabana H, Fagbamigbe AF. Decomposing the rural-urban disparities in overweight and obesity among women of reproductive age in Nigeria. BMC Womens Health. 2023 Dec 21;23(1):680.

96. Thapa R, Dahl C, Aung WP, Bjertness E. Urban–rural differences in overweight and obesity among 25–64 years old Myanmar residents: a cross-sectional, nationwide survey. BMJ Open. 2021 Mar 2;11(3):e042561.

97. Yeshaw Y, Kebede SA, Liyew AM, Tesema GA, Agegnehu CD, Teshale AB, et al. Determinants of overweight/obesity among reproductive age group women in Ethiopia: multilevel analysis of Ethiopian demographic and health survey. BMJ Open. 2020 Mar 1;10(3):e034963.

Table 1: Socio-economic characteristics of women of reproductive age in 40 low and middle-income countries, 2015 to 2022

Characteristics	Weighted frequency (%)	Underweight (95% CI)	Overweight & obesity (95% CI)
Region			
Africa	203,839 (19.5)	11.2 [11.1-11.3]	27.7 [27.5-27.9]
Asia	796,119 (75.2)	17.0 [16.9-17.1]	23.8 [23.7-23.9]
Others*	44,381 (4.2)	4.8 [4.6-5.0]	4.6 [4.5-4.6]
Place of residence			
Urban	365,235 (35.0)	10.5 [10.4-10.6]	36.7 [36.5-36.8]
Rural	679,104 (56.0)	17.4 [17.3-17.5]	20.7 [20.6-20.8]
Covered by health insurance (n = 993,367)			
No	738,086 (74.3)	15.1 [15.0-15.2]	26.6 [25.5-25.7]
Yes	255,281 (25.7)	15.9 [15.7-16.0]	25.3 [25.1-25.5]
Household wealth			
Poor	397,860 (38.1)	19.9 [19.7-20.0]	16.4 [16.2-16.5]
Middle	213,202 (20.4)	14.8 [14.6-14.9]	25.3 [25.1-25.5]
Rich	433,278 (41.5)	10.6 [10.5-10.7]	35.7 [35.6-35.9]
Age			
15-25 year	357,587 (34.2)	25.0 [24.9-25.2]	11.0 [10.9-11.1]
25-34 year	319,714 (30.6)	12.0 [11.9-12.1]	27.7 [27.5-27.8]
>=35 years	367,038 (35.2)	8.9 [8.8-9.0]	37.6 [37.4-37.7]
Educational status			
No formal education	237,726 (22.8)	15.9 [15.8-16.1]	21.3 [21.2-21.5]
Primary education	185,877 (17.8)	12.4 [12.2-12.5]	28.7 [28.5-28.9]
Secondary education	480,398 (46.0)	17.1 [17.0-17.2]	24.9 [24.7-25.0]
Higher education	140,336 (13.4)	11.9 [11.7-12.1]	31.0 [30.7-31.3]
Working status (n = 455,170)			
Non-working	252,205 (55.4)	13.9 [13.7-14.0]	28.4 [28.3-28.6]
Working	202,965 (44.6)	10.0 [9.9-10.1]	31.8 [31.6-32.0]
Marital status			
Never in union	254,214 (24.3)	26.3 [26.1-26.5]	11.3 [11.2-11.4]
Currently in union	733,430 (70.2)	11.7 [11.6-11.8]	30.0 [29.9-30.1]
Formerly in union	56,695 (5.5)	11.1 [10.9-11.4]	32.6 [32.2-33.0]
Media exposure			
No	446,154 (42.7)	18.1 [18.0-18.2]	18.5 [18.4-18.7]

Yes	598,186 (57.3)	13.1 [13.0-13.2]	31.1 [31.0-31.2]
Household head			
Male	839,056 (80.3)	15.6 [15.5-15.6]	25.0 [24.9-25.1]
Female	205,284 (19.7)	14.5 [14.3-14.6]	27.3 [27.0-27.4]

***Others**: Latin America, Caribbean, and Europe.

Table 2: Reproductive and health-related characteristics of women of reproductive age in 40 low and middle-income countries, 2015 to 2022

**Characteristics**	**Weighted frequency (%)**	**Underweight (95% CI)**	**Overweight & obesity (95% CI)**
Age at marriage (n =790,125)			
< 18 year	329,519 (41.7)	13.2 [13.1-13.4]	27.3 [27.2-27.5]
>= 18 year	460,606 (58.3)	10.6 [10.6-10.7]	32.1 [32.0-32.2]
History of pregnancy loss			
No	907,456 (86.9)	16.1 [16.0-16.2]	23.8 [23.7-23.9]
Yes	136,858 (13.1)	10.1 [9.9-10.3]	37.2 [36.9-37.4]
Parity			
Nullipara	310,076 (29.7)	24.7 [24.5-24.8]	12.7 [12.6-12.9]
Primipara	151,396 (14.5)	12.7 [12.5-12.8]	26.9 [26.6-27.1]
Multipara	479,261 (45.9)	10.8 [10.7-10.9]	32.4 [32.2-32.6]
Grand-multipara	103,607 (9.9)	11.8 [11.7-12.0]	30.2 [30.0-30.5]
Number of U5 children			
0	587,244 (56.2)	15.8 [15.7-15.9]	26.3 [26.2-26.4]
1	266,948 (25.6)	14.2 [14.1-14.3]	26.2 [26.0-26.3]
2+	190,148 (18.2)	15.6 [15.4-15.7]	21.8 [21.6-22.0]
Birth order			
1 to 3	548,478 (52.5)	11.2 [11.1-11.3]	31.1 [30.9-31.2]
>=4	495,862 (47.4)	19.8 [19.6-19.9]	19.6 [19.5-19.7]
Preceding birth interval (n = 580,547)			
< 33 months	274,304 (47.2)	12.1 [11.9-12.2]	29.3 [29.1-29.5]
>= 33 months	306,243 (52.8)	10.0 [9.9-10.1]	34.4 [34.3-34.6]
Anemia status (n = 951,070)			
No anemic	457,754 (48.1)	12.9 [12.8-13.0]	28.3 [28.2-28.5]
Anemia	493,316 (51.9)	18.1 [17.9-18.2]	21.7 [21.6-21.8]
Contraceptive use			
Not using	584,733 (56.0)	18.4 [18.3-18.5]	21.2 [21.1-21.3]

Using	459,607 (44.0)	11.2 [11.1-11.3]	31.2 [31.0-31.3]
Perceived distance to health facility (1,026,269)			
Not a big problem	752,281 (73.3)	14.8 [14.7-14.9]	27.0 [26.9-27.1]
Big problem	273,988 (26.7)	16.8 [16.6-16.9]	27.1[21.4-21.7]
Decision maker on women's health			
Woman alone	57,115 (5.5)	7.8 [7.6-8.0]	40.0 [39.6-40.4]
Jointly with partner	164,450 (15.7)	9.4 [9.3-9.6]	36.1 [35.8-36.3]
Others*	822,774 (78.8)	17.1 [17.0-17.1]	22.3 [22.3-22.4]
Toilet facility			
Unimproved	290,319 (27.8)	19.8 [19.6-19.9]	16.8 [16.7-16.9]
Improved	754,021 (72.2)	13.7 [13.6-13.7]	28.8 [28.7-28.9]
Drinking water source			
Unimproved	145,724 (14.0)	15.4 [15.2-15.5]	21.4 [21.2-21.6]
Improved	898,615 (86.0)	15.4 [15.3-15.4]	26.2 [26.1- 26.3]

*Others: husband only; someone else; other DHS category.

Table 3: Country-specific prevalence of underweight and overweight and obesity among women of reproductive age in 40 low- and middle-income countries, 2015 to 2022

Country	Survey year	Underweight	Overweight	Obesity	Total
Albania	2018	463 (4.3)	3108 (29.1)	1755 (16.4)	10668
Armenia	2016	210 (3.6)	1755 (29.9)	883 (15.0)	5877
Bangladesh	2018	2285 (11.5)	5037 (25.5)	1291 (6.5)	19786
Burkina Faso	2021	1030 (11.6)	1401 (15.8)	522 (5.9)	8852
Benin	2018	815 (10.1)	1362 (16.9)	732 (9.1)	8065
Burundi	2017	1520 (17.6)	575 (6.7)	153 (1.8)	8620
Cote d'Ivoire	2021	533 (7.4)	1533 (21.2)	867 (12.0)	7214
Cameroon	2018	395 (5.7)	1648 (23.9)	927 (13.4)	6892
Ethiopia	2016	3188 (21.1)	955 (6.3)	229 (1.5)	15123
Gabon	2019	458 (7.5)	1451 (23.8)	1517 (24.8)	6111
Ghana	2022	544 (7.0)	1917 (24.8)	1447 (18.7)	7724
Gambia	2019	762 (12.8)	1344 (22.6)	810 (13.6)	5952
Guinea	2021	480 (9.0)	970 (18.2)	434 (8.1)	5322
Guatemala	2015	688 (2.9)	7720 (31.9)	4813 (19.9)	24184
Haiti	2016	966 (10.1)	1998 (21.0)	1076 (11.3)	9530
India	2021	127250 (18.4)	121664 (17.6)	43643 (6.3)	692238
Jordan	2017	94 (1.3)	2722 (38.0)	2189 (30.6)	7155

Kenya	2022	1513 (9.2)	3995 (24.2)	2322 (14.1)	16486
Cambodia	2022	1024 (10.4)	2241 (22.8)	570 (5.8)	9833
Liberia	2019	208 (5.1)	1012 (24.8)	501 (12.3)	4083
Madagascar	2021	1680 (17.6)	1077 (11.3)	262 (2.7)	9553
Mali	2018	480 (9.4)	994 (19.4)	422 (8.2)	5119
Myanmar	2015	1898 (15.0)	2428 (19.2)	705 (5.6)	12640
Mauritania	2019	538 (7.3)	1980 (26.9)	1962 (26.6)	7363
Maldives	2016	724 (10.3)	2112 (30.2)	1373 (19.6)	6998
Malawi	2015	534 (6.7)	1284 (16.0)	429 (5.4)	8009
Mozambique	2022	398 (6.7)	953 (16.0)	452 (7.6)	5955
Nigeria	2018	1672 (11.3)	2703 (18.4)	1442 (9.8)	14730
Nepal	2022	961 (13.0)	1645 (22.3)	509 (6.9)	7370
Pakistan	2017	350 (8.0)	1329 (30.2)	925 (21.0)	4400
Rwanda	2019	405 (5.6)	1559 (21.5)	432 (6.0)	7265
Sierra Leone	2019	486 (6.5)	1517 (20.1)	599 (8.0)	7530
Chad	2015	2029 (17.9)	1085 (9.6)	263 (2.3)	11355
Tajikistan	2017	744 (7.0)	2597 (24.3)	1386 (13.0)	10669
Timor-Leste	2016	3180 (25.5)	1076 (8.6)	204 (1.6)	12451
Turkey	2018	250 (3.7)	1977 (29.5)	2026 (30.2)	6701
Tanzania	2022	696 (9.2)	1465 (19.4)	873 (11.5)	7570
Uganda	2016	481 (8.0)	1036 (17.2)	428 (7.1)	6012
Zambia	2018	97 (3.0)	875 (26.9)	1150 (35.4)	3251
Zimbabwe	2015	554 (5.7)	2251 (23.2)	1195 (12.3)	9684

Table 4: Measures of variations and model statistics summary for determinants of underweight and overweight and obesity among women of reproductive age in 40 LMICs, 2015 to 2022

**Measure of variation**	**Model 1**	**Model 2**	**Model 3**	**Model 4**
Cluster-level variance	0.44	0.27	0.23	31
Intra-class correlation	0.26	0.28	0.17	0.22
Explained variance (PCV)	Reference	0.39	0.47	0.29
Median odds ratio	1.72	1.35	1.24	1.44
**Model statistics summary**				
Log-likelihood	-242,243	-156,670	-242,128	-156,148

Deviance	484,486	313,340	482,256	312,296

Table 5: Determinants of underweight and overweight and obesity among women of reproductive age in 40 low and middle-income countries, 2015 to 2022

Characteristics	Underweight ARRR (95% CI)	Overweight and Obesity ARRR (95% CI)
Household wealth		
Poor	Ref	Ref
Middle	0.80 (0.78, 0.83)*	1.31 (1.29, 1.34)**
Rich	0.69 (0.67, 0.71)*	1.64 (1.61, 1.67)**
Age at marriage		
< 18 year	Ref	Ref
>= 18 year	0.93 (0.91-0.96)*	0.96 (0.94, 0.97)**
Age		
15-25 year	Ref	Ref
25-34 year	0.79 (0.77, 0.82)*	2.11 (2.05, 2.17)*
>=35 years	0.73 (0.70, 0.75)*	3.64 (3.53, 3.74)*
Educational status		
No formal education	Ref	Ref
Primary education	0.68 (0.66, 0.71)*	1.56 (1.52, 1.59)**
Higher education	0.65 (0.63, 0.67)*	1.91 (1.87, 1.96)**
Access to media		
No	Ref	Ref
Yes	0.79 (0.77, 0.81)*	1.60 (1.57, 1.63)**
Employment status		
Non-working	Ref	Ref
Working	0.89 (0.87, 0.91)*	0.85 (0.84, 0.86)*
Parity		
Nullipara	Ref	Ref
Primipara	0.99 (0.95, 1.04)	0.93 (0.90, 0.96)
Multipara	0.94 (0.89, 0.98)*	1.03 (0.99, 1.07)
Contraceptive use		
Not using	Ref	Ref
Using	0.94 (0.92, 0.97)*	1.22 (1.20, 1.25)**
Decision maker on women’s health		
Woman alone	Ref	Ref

Jointly with partner	1.09 (1.05, 1.13)*	0.94 (0.92, 0.96)*
Others+	1.08 (1.04, 1.12)*	0.86 (0.84, 0.88)*
Region		
Africa	Ref	Ref
Asia	1.46 (1.42, 1.50)*	1.00 (0.98, 1.02)
Others++	0.40 (0.37, 0.43)*	2.27 (2.20, 2.33)**
Place of residence		
Urban	0.80 (0.77, 0.83)*	1.73 (1.70, 1.76)*
Rural	Ref	Ref

+Others: husband only; someone else; other DHS category. ++Others: Latin America, Caribbean, and Europe. *P-value<0.05. **P-value<0.01. "Normal Weight" was the base category.

S1. STROBE Statement—checklist of items that should be included in reports of observational studies.

Figure 3

Click here to access/download;Figure;Figure 3.tif

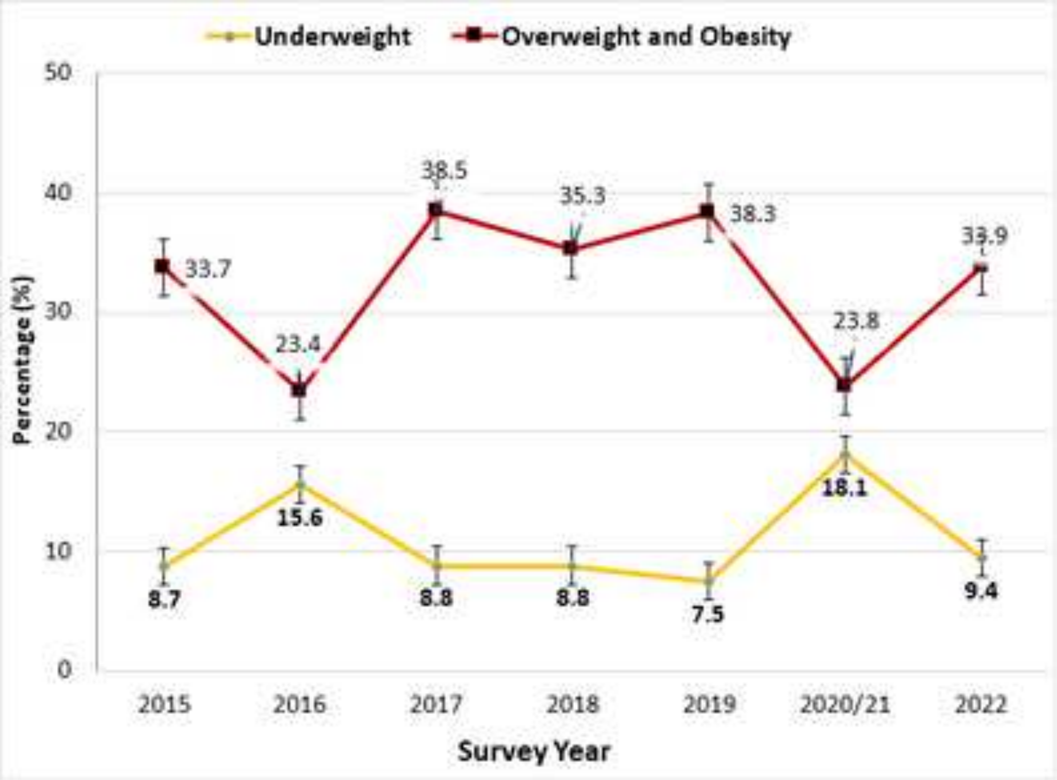

Figure 1

Click here to access/download;Figure;Figure 1.tif

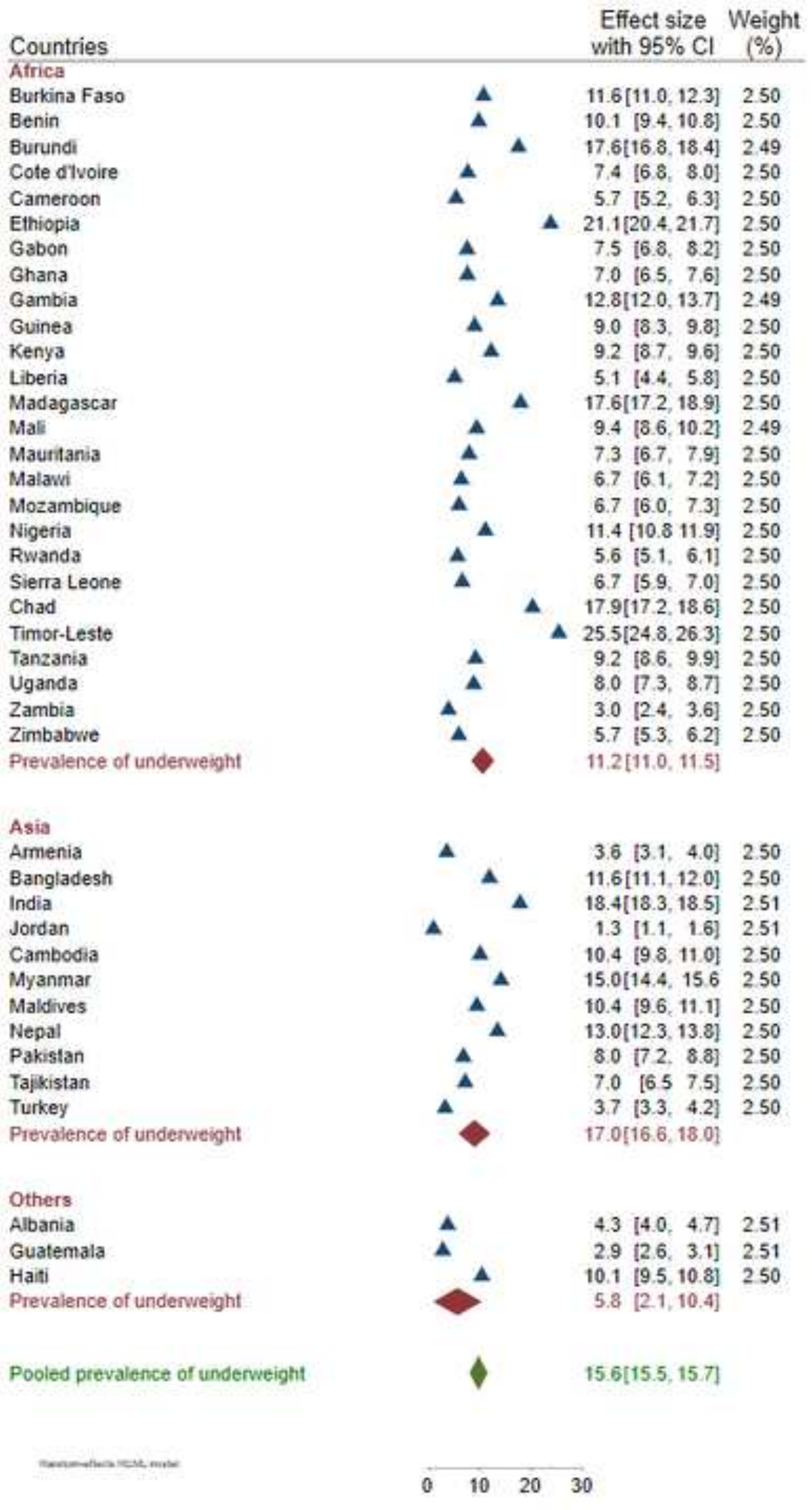

Figure 2

Click here to access/download;Figure;Figure 2.tif

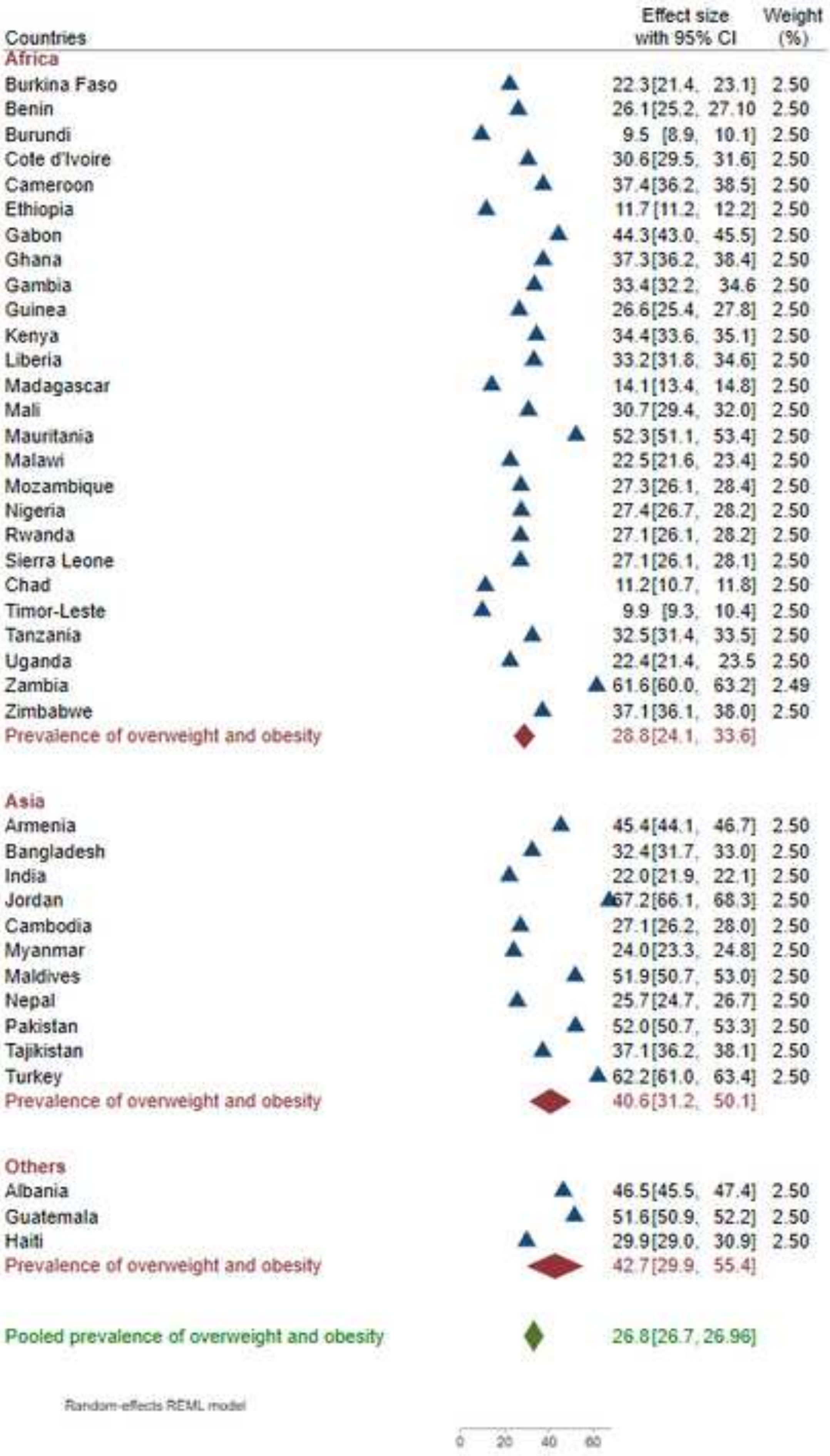

Other

Click here to access/download

Supporting Information

STROBE-checklist-v4-combined-PlosMedicine.docx

Level of overweight and obesity surpassed underweight among women in 40 low and middle-income countries: findings from a multilevel multinomial analysis of population survey data

Kusse Urmale Mare[1]*, Kebede Gemeda Sabo[1], Beriso Furo Wengoro[2], Begetayinoral Kussia Lahole[3]

Affiliations

[1]Department of Nursing, College of Medicine and Health Sciences, Samara University, Samara, Ethiopia

[2]Department of Biomedical Sciences, College of Medicine and Health Sciences, Samara University, Samara, Ethiopia

[3]Department of Midwifery, College of Medicine and Health Sciences, Arba Minch University, Arba Minch, Ethiopia

*__Corresponding author__: Kusse Urmale Mare; Email: kussesinbo@gmail.com

Formatted: Font color: Hyperlink

Formatted: Font color: Hyperlink

Abstract

Background: Despite continued global and local initiatives to address nutritional problems, low- and middle-income countries are facing both malnutrition and non-communicable diseases, with about 80% of non-communicable disease-related deaths. There is a dearth of recent evidence on the extent and determinants of underweight, overweight, and obesity in this region, which is essential for guiding intervention programs. Thus, this study intended to provide insights into the current level of ~~these abnormal nutritional indices~~malnutrition among women of reproductive age in low- and middle-income countries.

Methods: A secondary analysis of Demographic and Health Survey data from 40 low- and middle-income countries was performed using a weighted sample of 1,044,340 women of reproductive age. Forest plots and line ~~graphs~~graph were used to present the pooled and regional estimates of underweight~~,~~ and overweight~~,~~ and obesity. A multilevel multinomial logistic regression models were fitted to identify determinants of ~~abnormal nutritional indices~~malnutrition and models were compared based on the deviance and log-likelihood values. In the final model, statistical significance was determined using an adjusted relative risk ratio with the corresponding 95% confidence interval.

Results: The overall prevalence of obesity and overweight was 26.8% (95% CI: 26.7%-26.9%), with the highest rate in Jordan (67.2%) and lowest in Burundi (9.5%). Additionally, the level of underweight was found to be 15.6% [95% CI: 15.5%-15.7%], ranging from 1.3% in Jordan to 25.4% in Timor-Leste. Women of families with middle~~-~~ and ~~upper-income families~~rich wealth indexes, those aged 25-34 and 35-49, contraceptive users, those with primary and higher education, and

urban residents were more likely to be overweight or obese. In contrast, the results showed a lower likelihood of undernutrition among women in the households with middle~~-class~~ [ARRR (95% CI): 0.80 (0.78-0.83)] and rich ~~households~~wealth indexes [ARRR (95% CI): 0.69 (0.67-0.71)], those with primary [ARRR (95% CI): 0.68 (0.66-0.71)] and higher education [ARRR (95% CI): 0.65 (0.63-0.67)], and women with media access [ARRR (95% CI): 0.79 (0.77-0.81)].

Conclusion: ~~In conclusion, over~~Over a quarter of women of reproductive age in LMICs were overweight or obese, with underweight affecting 15.6%. It was also found that the levels of overweight and obesity were higher than the rate for underweight, with wide variations across the countries. Thus, efforts to improve the modifiable risks can have a positive impact on reducing ~~these abnormal nutritional indices.~~undernutrition and over-nutrition. Given the considerable variations between countries and survey periods, nutrition programs should also be tailored to the specific needs and cultural contexts of different regions within the study settings. Furthermore, the findings suggest the need for regular monitoring and evaluation of the existing nutritional interventions to assess their effectiveness.

Keywords: Low and middle-income countries, Underweight, Overweight, Obesity; Demographic and Health Survey, Determinants, Multilevel multinomial analysis, Women of reproductive age.

Background

Overweight and obesity are significant public health challenges in both developed and developing regions, disproportionately affecting women (40%) than men (35%) [1,2]. While overweight is marked by an excessive accumulation of body fat, obesity is a chronic and complex condition with substantial fat deposition. Both conditions pose varying degrees of health risks associated with

excessive body fat [1]. There are several linkages between obesity and health problems, including type 2 diabetes and heart disease, poor bone health, disturbed sleep, impaired reproductive function and mobility, and cancers of the reproductive organs [1,3,4].

Excessive weight gain in women is also associated with infertility, exacerbated polycystic ovary syndrome, heart disease, diabetes, and breast cancer due to changes in reproductive hormones [5,6]. ~~Furthermore, being~~Being overweight and obese during pregnancy is also linked with a higher risk of gestational diabetes and hypertension [7,8], and complications like postpartum hemorrhage, assisted delivery, and surgical site infections [9–11]. ~~Additionally~~Furthermore, maternal exposure to these nutritional indices during the prenatal period elevates the risk of congenital malformations, preterm birth, perinatal death, and macrosomia [5,12,13].

Globally, about 4.0 million deaths and 120 million disability-adjusted life-years are attributed to overweight and obesity [14]. In 2022, approximately 1 in 8 individuals worldwide were living with obesity and 2.5 billion adults aged 18 years and older were overweight, with 890 million affected by obesity [1]. Additionally, 43% of women worldwide are affected by overweight or obesity [1].

The prevalence of overweight and obesity is on the rise in developing countries alongside economic development, reduced physical activity, and a shift towards high-energy and fatty diets [15,16]. ~~For instance, among women, the level of overweight varies, with 18% in Brazil [17], 16% in Uganda [18], 10% in Nigeria[19], 15% in Cambodia [20], 50% in Tanzania [21], 37% in Zimbabwe [22], 60% in South Africa [23], in 12% in Ethiopia [24], and 13% in Colombia [25]. Likewise, obesity rates in women significantly vary across different regions, with 18% in Brazil [17], 6% in Uganda [18], 18% in Nigeria [19], 3% in Cambodia [20], 35% in South Africa [23], 3%~~

~~in Ethiopia [24], 29% in Colombia [25], and 9%, 10%, and 15% in Bangladesh, Nepal, and India respectively [26].~~For instance, among women, the level of overweight varies from 10% to 60% [17–25], while the prevalence of obesity ranges from 3% to 35% [17–20,23–26].

Previous studies showed that the factors associated with overweight and obesity include gender [8,27,28], age [8,27,29,30], marital status [26,27,29–32], wealth status [8,28–31], exposure to media [33,34], physical activities [28,34], alcohol consumption [31,34], snacking [31], education [29,30], occupation [30], contraceptive use [30], and place of residence [29,32].

Despite a continued effort of the International Fund for Agricultural Development and World Health Organization (WHO) to address the increasing rates of obesity and overweight, particularly in low- and middle-income countries (LMICs) [1,35], these regions are facing a double burden of malnutrition, where approximately 70% of the population are overweight or obese [35]. Additionally, these settings face an increased risk of non-communicable diseases (NCDs) such as diabetes and cardiovascular diseases, with about 80% of NCD-related deaths [35]. Despite these alarming figures, there is a lack of up-to-date evidence on the level and determinants of overweight and obesity in this region. Furthermore, previous studies in the region have utilized outdated DHS data, whereas our study aimed to bridge this gap by incorporating the most recent DHS datasets from the countries included. Thus, this study intended to provide insights into the current level of overweight and obesity among women of reproductive age (WRA) in LMICs using population survey data from 40 countries.

Formatted: Font: Calibri
Formatted: Font: Calibri
Formatted: Font: Calibri
Formatted: Font: Calibri
Formatted: Font: Calibri
Formatted: Font: Calibri
Formatted: Font: Calibri
Formatted: Font: Calibri
Formatted: Font: Calibri
Formatted: Font: Calibri
Formatted: Font: Calibri
Formatted: Font: Calibri
Formatted: Font: Calibri
Formatted: Font: Calibri
Formatted: Font: Calibri

Methods

Data source and participants

Data were obtained from demographic and health surveys conducted between 2015 and 2022 in 40 LMICs (https://dhsprogram.com/data/dataset-admin/index.cfm). Before inclusion, the survey dataset of each country was checked for the availability of body mass index (BMI) records, and countries with no data on this record were excluded from the analysis. After dropping records with missing observations and those with no unknown BMI data, a weighted sub-sample of 1,044,340 WRA (unweighted sample = 1,052,841) in all surveys was included in the analysis.

Variables and measurement

Dependent variable

The dependent ~~variables were underweight, overweight, and obesity which~~variable was ~~generated from the~~ BMI~~ data~~, measured as the ratio of the weight in kilograms to the square of the height in meters (kg/m^2). ~~Accordingly, BMI was classified based~~Based on the WHO cut-off point, BMI was classified as underweight, normal, overweight, and obese if the BMI is < 18.5 kg/m^2, 18.5-24.9 kg/m^2, 25-29.9 kg/m^2, and ≥ 30 kg/m^2, respectively [36]. For the analysis purpose, overweight and obesity were treated as one category.

Independent variables

Individual-level (level-1) variables included age, marital status, household wealth, health insurance coverage, women’s and partner's education, employment, marital status, media exposure, household head, age at marriage, history of pregnancy loss, parity, number of under-five children, birth order, preceding birth interval, anemia status, contraceptive use, decision

maker on women's health, source of drinking water, and toilet facility. While, region, place of residence, and distance to health facility were the community-level (level-2) variables.

Media exposure was constructed using the frequency of watching television, listening to the radio, and reading newspapers, which have three response options (i.e. not at all, less than once a week, and at least once a week). Using the DHS definition, women who indicated that they watched television, listened to the radio, or read the newspaper at least once a week were classified as having media exposure, while those who did not meet this criterion were classified as not having exposure.

Data management and analysis

Data cleaning and analysis were conducted using Stata software version 17. Before analysis, the presence of the outcome variable (i.e. BMI) in the DHS dataset of each country was confirmed, and all study variables were assessed for missing data. Records with missing observations and unknown BMI were dropped from the analysis. Finally, the datasets of 40 LMICs were appended and weighted to compensate for the non-representativeness of the sample and obtain reliable estimates and standard errors.

To account for the hierarchical structure of DHS design and the polytomous nature of the outcome variable, a multilevel multinomial logistic regression model was applied to determine the effects of independent variables on ~~abnormal nutritional indices.~~underweight and overweight and obesity. Initially, bivariable multilevel logistic regression analysis was done and variables with a p-value of less than 0.25 in this analysis were considered for the multivariable regression model.

Fixed-effect multilevel analysis

Four hierarchical models were independently fitted to select the model that best fits the data. To test the random variability in the intercept, an empty model i.e. a model without an independent variable was fitted. In the second and third models, the effect of individual and community level variables on abnormal BMI was respectively assessed, while the combined effect of both community and individual level variables on the outcome variable was simultaneously assessed in the fourth model.

In Model I (empty model), the likelihood of being underweight and overweight and obese is only a function of the clusters in which the women lived, which is indicated by a random intercept at the cluster level. In Model II (a model with ~~individual~~ level-1 variables), the probability of being underweight and overweight and obese was measured as a function of the cluster in which the women resided and individual-level characteristics. Model III (model with ~~community~~ level-2 variables) assessed the probability of malnutrition as a function of cluster and community-level variables, while Model IV (model with both ~~individual~~level-1 and ~~community~~ level-2 variables) examined the combined effect of both ~~individual~~level-1 and ~~community~~ level-2 variables on the occurrence of underweight, overweight~~,~~ and obesity.

After fitting these models, models were compared based on log-likelihood (LL) and deviance (i.e. -2*LL) values to select the one that best fits the data. For instance, model IV has the lowest deviance and highest LL values and is hence considered the best-fit model for the final analysis. A generalized variance inflation factor [GVIF = $VIF^{[1/(2*df)]}$] was used to determine the presence of collinearity between explanatory variables and this value was less than five for all variables

included in the final model, suggesting the non-existence of multi-collinearity. Finally, in the multivariable analysis, a p-value less than 0.05 and an adjusted relative risk ratio (ARRR) with the corresponding 95% confidence interval were used to identify determinants of underweight, overweight, and obesity.

Random-effect analysis (measures of variance)

Random variability in the level of underweight, overweight, and obesity across clusters was assessed by intra-class correlation coefficient (ICC), proportion change in variance (PCV), and median odds ratio (MOR). In this analysis, ICC indicates the total variance in the proportion of underweight, overweight, and obesity attributed to cluster differences. PCV (attributable percentage variability in the multilevel model) measured the differences in the prevalence of malnutrition attributed to community or individual-level characteristics. Heterogeneity in the risk of ~~abnormal nutritional indices~~underweight and overweight and obesity among women in different clusters was assessed by MOR. It was measured as the median value of the odds of the outcome variable (i.e. abnormal BMI) between women living in the area at the highest risk of abnormal BMI compared to those in the clusters at the lowest risk of the event.

Ethical approval

Since we used secondary data, permission to access the data was granted from the Measure DHS official Database via an online request at ~~http://www.dhsprogram.com.~~http://www.dhsprogram.com. The survey procedures were also approved by the Institutional Review Board of the host country and ICF International.

Additionally, written informed consent was initially obtained from the study participants during the collection of the survey data.

Results

Sociodemographic characteristics

Of 1,044,340 ~~WRA~~women of reproductive age, about three-fourths (75.2%) were from the Asia region, more than half (56.0%) resided in rural areas, and 433,278 (41.5%) were from rich households. Moreover, 357,587 (34.2) were between the ages of 15-24 years, less than one-quarter (22.8%) had no formal schooling, and 733,430 (70.2%) were currently in a union (Table 1).

In this analysis, the highest prevalence of underweight was observed in women from Asia (17.0%), rural settings (17.4%), poor households (19.9%), and younger age groups (35.0%). Conversely, women from the African region (27.7%), urban dwelling (36.7%), rich households (35.7%), older age groups (37.6%), and those with higher education (31.0%) had higher rates of overweight and obesity than their respective categories (Table 1).

Table 1: Socio-economic characteristics of women of reproductive age in 40 low and middle-income countries, 2015 to 2022

Reproductive and health-related characteristics

More than half (58.3%) of women were married at 18 years of age or older, 136,858 (13.1%) had experienced pregnancy loss, and 479,261 (45.9%) were multiparous. Additionally, about half (51.9%) of WRA were anemic, 459,607 (44.0%) were using contraceptive methods, and nearly a quarter (26.7%) reported that distance to the nearest health facility was a big problem.

The highest proportion of undernutrition was observed among nulliparous women (24.7%), women with a birth order of 4 or more (19.8%), anemic women (18.1%), and those not using contraception (18.4%). Conversely, multiparous women (32.4%), those with a birth interval of at least 33 months (34.4%), and women using contraceptives (31.2%) had comparatively higher levels of overweight and obesity. Furthermore, the prevalence of overweight and obesity was higher among women with a history of pregnancy loss (37.2%), those with a lower birth order (31.1%), and those who made their own healthcare decisions (40%) (Table 2).

Table 2: Reproductive and health-related characteristics of women of reproductive age in 40 low and middle-income countries, 2015 to 2022

Prevalence of underweight

Our result showed that the overall prevalence of underweight among WRA in LMICs was 15.6% [95% CI: 15.5%-15.7%], with large variations across the included countries. The highest level of undernutrition was observed in Timor-Leste (25.4%), Ethiopia (21.1%), Chad (17.9%), and Burundi (17.6%), while Jordan (1.3%), Guatemala (2.9%), and Zambia (3.0%) had the lowest rates ~~(Figure 1).~~ . Moreover, subgroup analysis revealed that Asia had the highest prevalence of underweight (17.0%), followed by Africa (11.2%) (Figure 1).

Figure 1: Pooled and national estimates of underweight among women of reproductive age in 40 low- and middle-income countries from 2015 to 2022.

Prevalence of overweight and obesity

The pooled prevalence of obesity and overweight among WRA in LMICs was 26.8% (95% IC: 26.7%-26.9%), of which overweight and obesity accounted for 18.8% [95% CI: 18.7%-18.9%] and

8.0% [95% CI: 7.9%-8.1], respectively. Higher rates were found in Jordan (67.2%), Turkey (62.2%), and Zambia (61.6%), while the lowest prevalence was noted in Burundi (9.5%) and Timor-Leste (9.9~~%) (Figure 2).~~%). Additionally, the analysis noted the highest prevalence of overnutrition among women in Latin America, Caribbean, and Europe (42.7%), followed by Africa (40.6%) (Figure 2).

Figure 2: Pooled and national estimates of overweight and obesity among women of reproductive age in 40 low- and middle-income countries from 2015 to 2022.

Of forty countries included in the analysis, the rate of overweight and obesity exceeded the prevalence of underweight in 35 countries. The exceptions were five countries (Burundi, Ethiopia, Madagascar, Chad, and Timor-Leste) where the highest level of undernutrition was observed (Table 3).

Table 3: Country-specific prevalence of underweight and overweight and obesity among women of reproductive age in 40 low- and middle-income countries, 2015 to 2022

Change in the level of underweight and overweight and obesity over survey years

Figure 3 shows the change in the level of ~~abnormal nutritional indices~~underweight and overweight and obesity across the survey years in 40 LMICs. This result indicated that the level of obesity and overweight surpassed that of underweight during all survey periods, with the peak prevalence in 2017 (38.5%) and 2019 (38.3%). Our analysis also showed a decline in undernutrition from 18.1% to 9.4% between 2020/21 and 2022, while the level of overweight and obesity rose by about 10%, from 23.8% to 33.9% during the same period. Overall, both undernutrition and overnutrition were slightly increased between 2015 and 2022 (Figure 3).

Figure 3: Change in the level of underweight and overweight and obesity among women of reproductive age in 40 low- and middle-income countries from 2015 to 2022.

Random effect result (measures of variations)

A random effect analysis result indicated that 44% of the variability in the level of malnutrition was due to differences at the cluster level (as indicated by the ICC value of model 1), while 31% of the total variability was explained by both individual and community-level factors (ICC value of model IV). In the final model (model IV), the PCV value also shows that 29% of the variation in malnutrition among WRA was explained by the effect of both individual and community-level covariates. Furthermore, the MOR of an empty model indicated the existence of heterogeneity in the magnitude of ~~abnormal nutritional indices~~underweight and overweight and obesity across clusters. This implies that WRA in the clusters with higher levels of malnutrition were 1.72 times more likely to suffer from malnutrition than those in the clusters with lower prevalence (Table 4).

Table 4: Measures of variations and model statistics summary for determinants of ~~abnormal nutritional indices~~underweight and overweight and obesity among women of reproductive age in 40 LMICs, 2015 to 2022

Determinants of underweight and overweight and obesity

The result showed that the risk of overweight and obesity was higher for WRA of households with middle~~-class~~ [ARRR (95% CI): 1.31 (1.29-1.34)] and rich ~~families~~wealth indexes [ARRR (95% CI): 1.64 (1.61-1.67)] and those aged 25-34 [ARRR (95% CI): 2.11 (2.05-2.17)] and 35-49 years [ARRR (95% CI): 3.64 (3.53-3.74)]. Moreover, contraceptive use [1.22 (1.20-1.25)], attending primary

[ARRR (95% CI): 1.56 (1.52-1.59)] and higher education [ARRR (95% CI): 1.91 (1.87-1.96)], and living in urban areas [ARRR (95% CI): 1.73 (1.70-1.76)] were associated with an increased risk overnutrition.

Our result also revealed that the likelihood of undernutrition was lower for women in the households with middle-class [ARRR (95% CI): 0.80 (0.78-0.83)] and rich householdswealth indexes [ARRR (95% CI): 0.69 (0.67-0.71)], those with primary [ARRR (95% CI): 0.68 (0.66-0.71)] and higher education [ARRR (95% CI): 0.65 (0.63-0.67)], and women with media access [ARRR (95% CI): 0.79 (0.77-0.81)]. Additionally, age, employment status, media exposure, women's involvement in healthcare decisions, parity, contraceptive use, and place of residence were significantly associated with undernutrition (Table 5).

Table 5: Determinants of abnormal nutritional indicesunderweight and overweight and obesity among women of reproductive age in 40 low and middle-income countries, 2015 to 2022

Discussion

This study assessed the level and determinants of underweight, overweight, and obesity among WRA in 40 LMICs, utilizing nationally representative data.. The pooled prevalence of overweight and obesity among women in LMIcs was 26.8% (95% CI: 26.7%-26.9%). Moreover, overweight affected 18.8% (95% CI: 18.7%-18.9%), while 8.0% (95% CI: 7.9%-8.1%) of women suffered from obesity. Higher rates of overweight and obesity were noted in Jordan (67.2%), Turkey (62.2%), and Zambia (61.6%), whereas Burundi (9.5%) and Timor-Leste (9.9%) exhibited the lowest levels. The current result is in line with prior studies in England (26%) [42] and Mali (26.9%) [41], but higher than the prevalence reported from the studies done in Ethiopia (15%) [24], Malawi (23%)

[37], Sub-Saharan Africa (22.6%) [38][38,39], and Uganda (22.4%) [39].[18]. Contrarily, our finding is lower compared to the studies conducted in South Africa (27%) [40][41], Nigeria (30%) [41][42], Cameroon (50%) [42][43], Egypt (85%) [43][44], Kenya (30%) [30], Zimbabwe (46.5%) [44][45], and Australia (60%) [45]. These disparities in the level of overweight and obesity across the studies[46]. These disparities could be attributed to divergent levels of economic development, urbanization promoting sedentary lifestyles, culture-specific food taboos and dietary practices, and variations in levels of physical activity and nutritional awareness across the study settings.

Formatted: Font: Calibri

Our findings also revealed that the pooled prevalence of underweight among women of childbearing age in LMICs was 15.6% [95% CI: 15.5%-15.7%], with substantial variability among the countries studied. This finding is lower than the rates reported in India (20%) [46], South Asia (24%) [47], and LMICs (42%) [47]. However, our finding is higher than reported in the previous studies in Bangladesh (12%) [48], Sierra Leone (6.7%) [49], global prevalence (9.7%) [50], Myanmar (14%) [51], Indonesia (14%) [52], and Botswana (10%) [53]. The observed variation in underweight prevalence among women in LMICs can be attributed to differences in socioeconomic status, disparities in education and health awareness, cultural dietary practices, regional food security discrepancies, and access to nutritional education.

Similar to the results of the studies done in Kenya [54], Bangladesh [55], India [56], Nepal [55], and SSA [57], our analysis revealed that women from middle-class and rich households exhibited a higher risk of overweight and obesity compared to their counterparts. Our study also found that belonging to wealthy families is associated with lower odds of being underweight compared to poorer families. This aligns with previous studies in various contexts [58–60] showing similar

associations. This could be because middle and wealthy households have easier access to calorie-dense diets, which can lead to increased calorie intake. Additionally, their higher socioeconomic status tends to correlate with lower physical activity levels and increased sedentary behavior. Furthermore, psychosocial factors such as stress associated with higher socioeconomic status can impact eating behaviors, further contributing to overweight and obesity [61].

In this analysis, women aged between 25-34 and 35-49 years were more likely to be overweight and obese than younger women. This aligns with the findings from the previous studies in different settings [62–65]. This might be due to biological changes and shifts in lifestyle as women age increases. Factors such as metabolic changes, reduced physical activity, and altered dietary habits might likely contribute to the higher risk of overweight and obesity observed in older women. Our finding also showed a lower risk of being underweight, overweight, and obese among women who married at the age of 18 or older. The possible justification could be that delaying marriage allows women more time to develop healthier lifestyle habits, such as improved diet and regular exercise. Additionally, it correlates with higher education and socio-economic status, factors known to contribute to better health outcomes and reduced risk of weight-related issues [66].

Our study found a higher risk of overweight and obesity among women with primary and higher education, alongside lower likelihood of underweight in these groups. These results align with previous studies across different settings [67–69]. The possible reason might be that higher educational attainment may contribute to better weight management practices by providing increased access to resources and knowledge about nutrition and healthy lifestyles. Furthermore, higher educational attainment often leads to greater economic stability, which can improve

access to healthier food options. This improved access may contribute to a lower risk of underweight among women with higher education levels. However, on the other side, it can increase the risk of overweight and obesity due to potential changes in dietary habits and lifestyle factors associated with socio-economic status [70].

Access to mass media was also identified as another significant factor linked with abnormal nutritional indices. The study found that media access was associated with a higher risk of obesity and overweight and a lower risk of underweight. Similarly, previous studies in both developed and developing nations [71–74] have reported comparable associations. Potential explanations include the promotion of unhealthy foods and sedentary behaviors via media, which can contribute to weight gain and obesity. Unrealistic body ideals portrayed in the media may also lead to body image issues and disordered eating, impacting both obesity and underweight trends. However, access to media also creates an opportunity for health literacy on dietary practices, potentially reducing the risks of abnormal nutrition, and influencing cultural norms regarding dietary habits and weight management practices [75].

Our analysis found that employed women had a higher likelihood of abnormal nutritional indices compared to their non-employed counterparts. Studies conducted in Malaysia, Ethiopia, and Mexico have reported similar findings [76–78]. The possible explanation could be that employed women experience higher stress levels and time constraints, leading to irregular eating habits and reliance on convenient but less nutritious food choices compared to their counterparts.

Similar to studies in both high- and low-income countries [23,79–81], our analysis showed that multiparous women have a higher risk of overweight and obesity than primipara and nullipara

women. This could be due to the cumulative effect of weight gain across successive pregnancies, where weight gained during each pregnancy is not completely lost before the next pregnancy. Additionally, the demands of childcare and family responsibilities may reduce opportunities for physical activity and healthy meal planning, further contributing to the higher likelihood of overweight and obesity among multipara [79].

Contraceptive use was also found to be associated with abnormal nutritional indices. The study showed that women using contraceptives are more likely to be overweight or obese compared to non-users. The current findings align with previous studies [82–84]. This can be explained by the fact that hormonal contraceptives alter hormone levels, potentially influencing metabolism, fat deposition, and appetite regulation, which promote weight gain. Additionally, reduced concern about pregnancy-related weight changes among contraceptive users may lead to decreased attention to diet and exercise, further contributing to higher rates of overweight and obesity [85,86].

Furthermore, our analysis revealed that women whose healthcare decisions relied solely on their husbands' decisions or who made joint decisions with their partners were more likely to be underweight compared to other women. Consistently, previous studies have shown similar associations [87–89]. This might be because societal norms and gender roles limit women's autonomy in practicing healthy behaviors and accessing resources like nutritious food and healthcare. These constraints, compounded by cultural expectations and lower social support within decision-making frameworks, ultimately contribute to inadequate nutrition and healthcare utilization, leading to poorer health outcomes such as underweight.

In addition, our analysis revealed that compared to African women, women in Asia, Latin America, the Caribbean, and Europe had a higher risk of being overweight and obese. Additionally, women in Asia also faced a double burden, with a significant risk of underweight alongside overweight and obesity. Likewise, previous studies in LMICs have shown similar trends in overweight and obesity [90–92]. The possible reason could be that in rapidly urbanizing and economically growing regions like Asia, Latin America, the Caribbean, and Europe, increased sedentary behavior, higher consumption of processed foods, and reduced physical activity contribute to rising obesity rates. Conversely, in less urbanized areas in Asia and Africa, traditional diets and more active lifestyles may help maintain a lower risk of obesity [93].

Urban residence was found as a significant factor associated with increased risk of overweight and obesity among women. Women residing in urban areas exhibited a higher risk of overweight and obesity compared to their rural counterparts. This is consistent with the previous studies in SSA [57], Nigeria [94], Myanmar [95], and Ethiopia [96], which demonstrated a higher likelihood of overnutrition among urban residents. The possible explanation could be that in rural areas, women's active engagement in agriculture helps limit weight gain compared to urban women. Additionally, rural lifestyles generally involve less exposure to sedentary behavior, modern transportation reduced physical activity, and consumption of high-fat, energy-dense foods typical of urban settings Our findings also revealed that the prevalence of underweight among WRA in LMICs was 15.6% [95% CI: 15.5%-15.7%], with substantial variations among the countries studied. This finding is lower than the rates reported in India (20%) [47], South Asia (24%) [48], and LMICs (42%) [48]. However, our finding is higher than that reported in the studies done in Bangladesh (12%) [49], Sierra Leone (6.7%) [50], global prevalence (9.7%) [51], Myanmar (14%) [52],

Indonesia (14%) [53], and Botswana (10%) [54]. These variations can be attributed to differences in socioeconomic status, disparities in education and health awareness, cultural dietary practices, regional food security discrepancies, and access to nutritional education across different settings.

Similar to the results of the studies done in Kenya [55], Bangladesh [56], India [57], Nepal [56], and SSA [58], our analysis revealed that women from households with middle and rich wealth indexes exhibited a higher risk of overweight and obesity compared to their counterparts. Our study also found that belonging to wealthy families is associated with lower odds of being underweight compared to women of families with poor wealth index. This aligns with previous studies in various contexts [59–61] showing similar associations. This could be because women of households with middle and rich wealth indexes have relatively easier access to calorie-dense diets, which can lead to increased calorie intake. Additionally, their higher socioeconomic status tends to correlate with lower physical activity levels and increased sedentary behavior. Furthermore, psychosocial factors like stress associated with higher socioeconomic status can impact eating behaviors, further contributing to overweight and obesity [62].

In this analysis, women aged between 25-34 and 35-49 years were more likely to be overweight and obese than younger women. This aligns with the findings from the previous studies in different settings [63–66]. This might be due to biological changes and shifts in lifestyle as women age increases. Factors such as metabolic changes, reduced physical activity, and altered dietary habits might likely contributed to the observed result. Our finding also showed a lower risk of underweight, overweight and obesity among women who married at the age of 18 or older. The possible justification could be that delaying marriage allows women more time to develop healthier lifestyle habits, such as improved diet and regular exercise. Additionally, it correlates

with higher education and socio-economic status, factors known to contribute to better health outcomes and reduced risk of weight-related issues [67].

Our study found a higher risk of overweight and obesity among women with primary and higher education, alongside lower likelihood of being underweight in these groups. These results align with previous studies across different settings [68–70]. The possible reason might be that higher educational attainment may contribute to better weight management practices by providing increased access to resources and knowledge about nutrition and healthy lifestyles. Furthermore, higher educational attainment often leads to greater economic stability, which can improve access to healthier food options. This improved access may contribute to a lower risk of underweight among women with higher education levels. However, on the other side, it can increase the risk of overweight and obesity due to potential changes in dietary habits and sedentary lifestyles (always in cars, with maid in houses, low levels of physical activity) associated with socio-economic status like [71].

Access to mass media was also identified as a significant factor linked with both undernutrition and overnutrition. The study found that media access was associated with a higher risk of obesity and overweight and a lower risk of underweight. Similarly, previous studies in both developed and developing nations [72–75] have reported comparable associations. Potential explanations include the promotion of unhealthy foods and sedentary behaviors via media, which can contribute to weight gain and obesity. Unrealistic body ideals portrayed in the media may also lead to body image issues and disordered eating, impacting both obesity and underweight trends. However, access to media also creates an opportunity for health literacy on dietary practices,

potentially reducing the risks of malnutrition, and influencing cultural norms regarding dietary habits and weight management practices [76].

We found that employed women had a lower likelihood of undernutrition and overnutrition compared to their non-employed counterparts. Studies conducted in Malaysia, Ethiopia, and Mexico have reported similar findings [77–79]. The possible explanation could be that employed women experience higher stress levels and time constraints, leading to irregular eating habits and reliance on convenient but less nutritious food choices compared to their counterparts.

Similar to studies in both high- and low-income countries [23,80–82], our analysis showed that multiparous women have a higher risk of overweight and obesity than primipara and nullipara women. This could be due to the cumulative effect of weight gain across successive pregnancies, where weight gained during each pregnancy is not completely lost before the next pregnancy. Additionally, the demands of childcare and family responsibilities may reduce opportunities for physical activity and healthy meal planning, further contributing to the higher likelihood of overweight and obesity among multipara [80].

Contraceptive use was also found to be associated with overweight and obesity. The study showed that women using contraceptives are more likely to be overweight or obese compared to non-users. The current findings align with previous studies [83–85]. This can be explained by the fact that hormonal contraceptives alter hormone levels, potentially influencing metabolism, fat deposition, and appetite regulation, which promote weight gain. Additionally, reduced concern about pregnancy-related weight changes among contraceptive users may lead to

decreased attention to diet and exercise, further contributing to a higher risk of overweight and obesity [86,87].

Furthermore, our analysis revealed that women whose healthcare relied solely on their husbands' decisions or who made joint decisions with their partners were more likely to be underweight compared to other women. Consistently, previous studies have shown similar associations [88–90]. This might be because societal norms and gender roles limit women's autonomy in practicing healthy behaviors and access to nutritious food and healthcare. These constraints, compounded by cultural expectations and lower social support within decision-making frameworks, ultimately contribute to inadequate nutrition and healthcare utilization, leading to poorer health outcomes such as underweight.

In addition, it was found that compared to African women, women in Asia, Latin America, the Caribbean, and Europe had a higher risk of being overweight and obese. Additionally, women in Asia also faced a double burden of malnutrition, with a significant risk of underweight and overweight and obesity. Likewise, previous studies in LMICs have shown similar trends in overweight and obesity [91–93]. The possible reason could be that in rapidly urbanizing and economically growing regions like Asia, Latin America, the Caribbean, and Europe, increased sedentary behavior, higher consumption of processed foods, and reduced physical activity contribute to rising obesity rates. Conversely, in less developed settings like Asia and Africa, traditional diets and more active lifestyles may help maintain a lower risk of obesity [94].

Urban residence was also significantly associated with an increased risk of overweight and obesity. Women residing in urban areas exhibited a higher risk of overweight and obesity

compared to their rural counterparts. This is consistent with the previous studies in SSA [58], Nigeria [95], Myanmar [96], and Ethiopia [97]. The possible explanation could be that in rural areas, women's active engagement in agriculture helps limit weight gain compared to urban women. Additionally, rural lifestyles generally involve less exposure to sedentary behavior, modern transportation, reduced physical activity, and consumption of high-fat, energy-dense foods typical of urban settings [1].

Strengths and limitations

The strengths of this analysis lie in the use of ~~a~~ recent population survey data from 40 countries, and the application of advanced statistical methods i.e. multilevel multinomial modeling to account for the nested structure of the DHS data and the polytomous nature of the outcome variable. However, it is important to acknowledge certain limitations when interpreting the study's findings. These include potential over or underestimation of estimates due to a large number of missing observations (274,180 missing), and differences in the survey year, number of countries and sample sizes ~~in~~across the regions included in the analysis might affect the regional estimates. The use of BMI for assessing nutritional status does not differentiate between excess fat and bone mass and does not provide information about the distribution of body fat. Additionally, the study did not examine the effect of physical activity, dietary patterns, and household food security status on the outcome variable since DHS does not gather data on these variables.

Conclusion

~~In conclusion, the~~The pooled prevalence of obesity and overweight among WRA in LMICs was 26.8%, while underweight affected 15.6%. Our analysis also revealed that the levels of overweight and obesity were higher than the rate for underweight, with wide variations across the countries and survey periods. Moreover, the result showed that factors like household wealth, maternal education, age, place of residence, contraceptive use, media access, healthcare decisions, and employment status bidirectionally influenced both underweight and overweight and obesity. Efforts to improve household wealth, maternal literacy and healthcare, and employment opportunities for women can have a positive impact on reducing both underweight and overweight and obesity. Given the considerable variations between countries and survey time, nutrition programs should be tailored to the specific needs and cultural contexts of different regions within the study settings. Furthermore, the study's findings suggest the need for regular monitoring and evaluation of the existing nutritional interventions to assess their effectiveness.

Abbreviations

ARRR: Adjusted Relative Risk Ratio; AIC: BMI: Body Mass Index; DHS: Demographic and Health Survey; GVIF: Generalized Variance Inflation Factor; ICC: Intra-class correlation; LL: Log-likelihood; LMICs: Lower- and Middle-Income Countries; MOR: Median Odds Ratio; NCD: Non-communicable Diseases; PCV: Proportion Change in Variance; SSA: Sub-Saharan Africa; WHO: World Health Organization; WRA: Women of Reproductive Age.

~~**Declarations**~~

~~**Consent for publication**~~

~~Not applicable~~

~~**Availability of data and materials**~~

~~The data used and analyzed in this study can be accessed from the DHS website (https://dhsprogram.com/data/dataset_admin/index.cfm).~~

~~**Competing interest**~~

~~The authors have declared that no competing interests exist.~~

~~**Funding**~~

~~No specific fund was received for this work.~~

~~**Authors' contributions**~~

~~KUM and BKL were involved in the conception and design of the study, acquisition, and analysis of data, interpretation of the results, and drafting of the manuscript. KGS and BFW participated in the design of the study, data acquisition, and drafting of the manuscript. KUM and BKL extensively revised the manuscript. All authors read and approved the final manuscript and agreed to take responsibility for the contents of this work.~~

Acknowledgments

The authors thank ICF International for granting access to the dataset used in this study.

References

1. Obesity and overweight [Internet]. [cited 2024 Jun 18]. Available from: https://www.who.int/news-room/fact-sheets/detail/obesity-and-overweight

2. Chong B, Jayabaskaran J, Kong G, Chan YH, Chin YH, Goh R, et al. Trends and predictions of malnutrition and obesity in 204 countries and territories: an analysis of the Global Burden of Disease Study 2019. eClinicalMedicine [Internet]. 2023 Mar 1 [cited 2024 Jun 18];57. Available from: https://www.thelancet.com/journals/eclinm/article/PIIS2589-5370(23)00027-5/fulltext

3. Fruh SM. Obesity: Risk factors, complications, and strategies for sustainable long-term weight management. J Am Assoc Nurse Pract. 2017 Oct;29(S1):S3.

4. Cefalu WT, Bray GA, Home PD, Garvey WT, Klein S, Pi-Sunyer FX, et al. Advances in the Science, Treatment, and Prevention of the Disease of Obesity: Reflections From a Diabetes Care Editors' Expert Forum. Diabetes Care. 2015 Jul 14;38(8):1567–82.

5. Overweight and Obesity - Obesity and Women's Health | NHLBI, NIH [Internet]. 2022 [cited 2024 Jun 18]. Available from: https://www.nhlbi.nih.gov/health/overweight-and-obesity/women

6. Obesity: Health consequences of being overweight [Internet]. [cited 2024 Jun 18]. Available from: https://www.who.int/news-room/questions-and-answers/item/obesity-health-consequences-of-being-overweight

7. Hossain MB, Khan JR, Gupta RD. Role of hypertension in the association of overweight and obesity with diabetes among adults in Bangladesh: a population-based, cross-sectional nationally representative survey. BMJ Open. 2021 Jul 1;11(7):e050493.

8. Prevalence and factors associated with overweight and obesity among adults in Hawassa city, southern Ethiopia: a community based cross-sectional study | BMC Obesity | Full Text

Formatted: Justified

[Internet]. [cited 2024 Jun 18]. Available from: https://bmcobes.biomedcentral.com/articles/10.1186/s40608-019-0227-7

9. Martins I, Ayres-de-Campos D. 41 - Assisted vaginal delivery in obese women. In: Mahmood T, Lim CC, editors. Handbook of Obesity in Obstetrics and Gynecology [Internet]. Academic Press; 2023 [cited 2024 Jun 21]. p. 353–6. Available from: https://www.sciencedirect.com/science/article/pii/B9780323899048000202

10. Butwick AJ, Abreo A, Bateman BT, Lee HC, El-Sayed YY, Stephansson O, et al. The Effect of Maternal Body Mass Index on Postpartum Hemorrhage. Anesthesiology. 2018 Apr;128(4):774–83.

11. Mitchell CJ, Adkins L, Tucker A, Brown H, Siegel A, Dotter-Katz S. Impact of Excess Weight Gain on Risk of Postpartum Infection in Class III Obesity. AJP Rep. 2020 Jul;10(3):e213–6.

12. Being overweight in pregnancy and after birth [Internet]. RCOG. [cited 2024 Jun 18]. Available from: https://www.rcog.org.uk/for-the-public/browse-our-patient-information/being-overweight-in-pregnancy-and-after-birth/

13. Obesity and Pregnancy | ACOG [Internet]. [cited 2024 Jun 18]. Available from: https://www.acog.org/womens-health/faqs/obesity-and-pregnancy

14. null null. Health Effects of Overweight and Obesity in 195 Countries over 25 Years. N Engl J Med. 2017;377(1):13–27.

15. Obesity [Internet]. [cited 2024 Jun 18]. Available from: https://www.who.int/health-topics/obesity

16. Weight of nations: a socioeconomic analysis of women in low- to middle-income countries - ScienceDirect [Internet]. [cited 2024 Jun 18]. Available from: https://www.sciencedirect.com/science/article/pii/S0002916523020658?via%3Dihub

17. Araújo FG, Velasquez-Melendez G, Felisbino-Mendes MS. Prevalence trends of overweight, obesity, diabetes and hypertension among Brazilian women of reproductive age based on sociodemographic characteristics. Health Care Women Int. 2019 Apr 3;40(4):386–406.

18. Yaya S, Ghose B. Trend in overweight and obesity among women of reproductive age in Uganda: 1995–2016. Obes Sci Pract. 2019 ~~Aug~~Jul 11;5(4):312–23.

19. Tagbo SO, Abebe D, Oguoma VM. Overweight and obesity among non-pregnant women of reproductive age in Nigeria: findings from the 2008–2018 Nigerian Demographic and Health Survey. Public Health. 2021 Sep 1;198:348–57.

20. Um S, Yom A, Muir JA, Sopheab H. Overweight and obesity among women at reproductive age in Cambodia: Data analysis of Cambodia Demographic and Health Survey 2014. PLOS Glob Public Health. 2023 Mar 31;3(3):e0001768.

21. Mosha D, Paulo HA, Mwanyika-Sando M, Mboya IB, Madzorera I, Leyna GH, et al. Risk factors for overweight and obesity among women of reproductive age in Dar es Salaam, Tanzania. BMC Nutr. 2021 Jul 16;7(1):37.

22. Mukora-Mutseyekwa F, Zeeb H, Nengomasha L, Kofi Adjei N. Trends in Prevalence and Related Risk Factors of Overweight and Obesity among Women of Reproductive Age in Zimbabwe, 2005–2015. Int J Environ Res Public Health. 2019 Jan;16(15):2758.

23. Nglazi MD, Ataguba JEO. Overweight and obesity in non-pregnant women of childbearing age in South Africa: subgroup regression analyses of survey data from 1998 to 2017. BMC Public Health. 2022 Feb 25;22(1):395.

24. Mengesha Kassie A, Beletew Abate B, Wudu Kassaw M. Education and prevalence of overweight and obesity among reproductive age group women in Ethiopia: analysis of the 2016 Ethiopian demographic and health survey data. BMC Public Health. 2020 Jul 31;20(1):1189.

25. Kordas K, Centeno ZYF, Pachón H, Soto AZJ. Being Overweight or Obese Is Associated with Lower Prevalence of Anemia among Colombian Women of Reproductive Age. J Nutr. 2013 Feb 1;143(2):175–81.

26. Balarajan Y, Villamor E. Nationally Representative Surveys Show Recent Increases in the Prevalence of Overweight and Obesity among Women of Reproductive Age in Bangladesh, Nepal, and India, ,. J Nutr. 2009 Nov 1;139(11):2139–44.

27. Ajayi IO, Adebamowo C, Adami HO, Dalal S, Diamond MB, Bajunirwe F, et al. Urban–rural and geographic differences in overweight and obesity in four sub-Saharan African adult populations: a multi-country cross-sectional study. BMC Public Health. 2016 Oct 28;16(1):1126.

28. Shayo GA, Mugusi FM. Prevalence of obesity and associated risk factors among adults in Kinondoni municipal district, Dar es Salaam Tanzania. BMC Public Health. 2011 May 23;11(1):365.

29. Delbiso TD, Rodriguez-Llanes JM, Altare C, Masquelier B, Guha-Sapir D. Health at the borders: Bayesian multilevel analysis of women's malnutrition determinants in Ethiopia. Glob Health Action. 2016 Jul 4;9:10.3402/gha.v9.30204.

30. Mkuu RS. Prevalence and Predictors of Overweight and Obesity Among Kenyan Women. Prev Chronic Dis [Internet]. 2018 [cited 2024 Jun 18];15. Available from: https://www.cdc.gov/pcd/issues/2018/17_0401.htm

31. Dagne S, Gelaw YA, Abebe Z, Wassie MM. <p>Factors associated with overweight and obesity among adults in northeast Ethiopia: a cross-sectional study</p>. Diabetes Metab Syndr Obes. 2019 Mar 22;12:391–9.

32. Janghorbani M, Amini M, Willett WC, Gouya MM, Delavari A, Alikhani S, et al. First Nationwide Survey of Prevalence of Overweight, Underweight, and Abdominal Obesity in Iranian Adults. Obesity. 2007;15(11):2797–808.

33. Musaiger A, Al-Mannai M. Association between exposure to media and body weight concern among female university students in five Arab countries: A preliminary cross-cultural study. J Biosoc Sci. 2013 Jun 12;46:1–8.

34. Darebo T, Mesfin A, Gebremedhin S. Prevalence and factors associated with overweight and obesity among adults in Hawassa city, southern Ethiopia: a community based cross-sectional study. BMC Obes. 2019 Mar 4;6:8.

35. We must do more to curb obesity and overweight in low- and middle-income countries [Internet]. IFAD. [cited 2024 Jun 18]. Available from: https://www.ifad.org/en/web/latest/-/we-must-do-more-to-curb-obesity-and-overweight-in-low-and-middle-income-countries

36. Malnutrition in women [Internet]. [cited 2024 Nov 22]. Available from: https://www.who.int/data/nutrition/nlis/info/malnutrition-in-women

37. Mndala L, Kudale A. Distribution and social determinants of overweight and obesity: a cross-sectional study of non-pregnant adult women from the Malawi Demographic and Health Survey (2015-2016). Epidemiol Health. 2019;41:e2019039.

38.38. Double burden of malnutrition and associated factors among women of reproductive age in sub-Saharan Africa: a multilevel multinomial logistic regression analysis | BMJ Open [Internet]. [cited 2024 Dec 20]. Available from: https://bmjopen.bmj.com/content/14/2/e073447.long

39. Neupane S, K. C. P, Doku DT. Overweight and obesity among women: analysis of demographic and health survey data from 32 Sub-Saharan African Countries. BMC Public Health. 2015 Dec;16(1):1–9.

39. Yaya S, Ghose B. Trend in overweight40. Double burden of malnutrition and obesityassociated factors among women of reproductive age in Uganda: 1995–2016. Obes Sci Pract. 2019 Jul 11;5(4):312–23. sub-Saharan Africa: a multilevel multinomial logistic regression analysis | BMJ Open [Internet]. [cited 2024 Dec 20]. Available from: https://bmjopen.bmj.com/content/14/2/e073447.long

Formatted: Justified

~~40~~41. Sartorius B, Veerman LJ, Manyema M, Chola L, Hofman K. Determinants of Obesity and Associated Population Attributability, South Africa: Empirical Evidence from a National Panel Survey, 2008-2012. PLoS ONE. 2015 Jun 10;10(6):e0130218.

~~41~~42. Chukwuonye II, John, Ohagwu, Ejiji, Imoh, Chuku, et al. Prevalence of overweight and obesity in adult Nigerians – a systematic review. Diabetes Metab Syndr Obes Targets Ther. 2013 Jan;43.

~~42~~43. Simo LP, Agbor VN, Temgoua FZ, Fozeu LCF, Bonghaseh DT, Mbonda AGN, et al. Prevalence and factors associated with overweight and obesity in selected health areas in a rural health district in Cameroon: a cross-sectional analysis. BMC Public Health. 2021 Mar 10;21(1):475.

~~43~~44. Reyes Matos U, Mesenburg MA, Victora CG. Socioeconomic inequalities in the prevalence of underweight, overweight, and obesity among women aged 20–49 in low- and middle-income countries. Int J Obes. 2020 Mar;44(3):609–16.

~~44~~45. Mangemba NT, San Sebastian M. Societal risk factors for overweight and obesity in women in Zimbabwe: a cross-sectional study. BMC Public Health. 2020 Jan 28;20(1):103.

~~45~~46. Overweight and obesity, Summary [Internet]. Australian Institute of Health and Welfare. 2024 [cited 2024 Jun 22]. Available from: https://www.aihw.gov.au/reports/overweight-obesity/overweight-and-obesity/contents/overweight-and-obesity

~~46~~47. Pengpid S, Peltzer K. Prevalence and correlates of underweight and overweight/obesity among women in India: results from the National Family Health Survey 2015–2016. Diabetes Metab Syndr Obes Targets Ther. 2019 May;Volume 12:647–53.

~~47~~48. Victora CG, Christian P, Vidaletti LP, Gatica-Domínguez G, Menon P, Black RE. Revisiting maternal and child undernutrition in low-income and middle-income countries: variable progress towards an unfinished agenda. Lancet Lond Engl. 2021 Apr 10;397(10282):1388–99.

~~48~~49. Hasan MdM, Ahmed S, Soares Magalhaes RJ, Fatima Y, Biswas T, Mamun AA. Double Burden of Malnutrition Among Women of Reproductive Age in Low- and Middle-Income Countries: Progress Achieved and Opportunities for Meeting the Global Target. Curr Dev Nutr. 2020 Jun 1;4:nzaa053_044.

~~49~~50. Ikoona EN, Toure MA, Njenga A, Namulemo L, Kaluya R, Kamara K, et al. Prevalence and factors associated with underweight among 15–49-year-old women in Sierra Leone: a secondary data analysis of Sierra Leone demographic health survey of 2019. BMC Womens Health. 2023 Apr 22;23(1):192.

~~50~~51. Trends in adult body-mass index in 200 countries from 1975 to 2014: a pooled analysis of 1698 population-based measurement studies with 19·2 million participants. The Lancet. 2016 Apr 2;387(10026):1377–96.

~~51~~52. Hong SA, Peltzer K, Lwin KT, Aung LS. The prevalence of underweight, overweight and obesity and their related socio-demographic and lifestyle factors among adult women in Myanmar, 2015-16. PLOS ONE. 2018 Mar 16;13(3):e0194454.

~~52~~53. Hanandita W, Tampubolon G. The double burden of malnutrition in Indonesia: Social determinants and geographical variations. SSM - Popul Health. 2015 Dec 1;1:16–25.

~~53~~54. Letamo G, Navaneetham K. Prevalence and Determinants of Adult Under-Nutrition in Botswana. PLOS ONE. 2014 Jul 23;9(7):e102675.

~~54~~55. Haregu TN, Mohamed SF, Muthuri S, Khayeka-Wandabwa C, Kyobutungi C. Body mass index and wealth index: positively correlated indicators of health and wealth inequalities in Nairobi slums. Glob Health Epidemiol Genomics. 2018 Jun 4;3:e11.

~~55~~56. Bishwajit G. Household wealth status and overweight and obesity among adult women in Bangladesh and Nepal. Obes Sci Pract. 2017;3(2):185–92.

~~56~~57. Let S, Tiwari S, Singh A, Chakrabarty M. Spatiotemporal change in wealth-based inequalities in overweight/obesity among women of reproductive age in India, 2015–2021. Clin Epidemiol Glob Health. 2023 Nov 1;24:101458.

~~57~~58. Atsu P, Mohammed A, Adu C, Aboagye RG, Ahinkorah BO, Seidu AA. Residence-based inequalities in overweight/obesity in sub-Saharan Africa: a multivariate non-linear decomposition analysis. Trop Med Health. 2024 Apr 7;52(1):29.

~~58~~59. Rahman A, Sathi NJ. Sociodemographic Risk Factors of Being Underweight Among Ever-Married Bangladeshi Women of Reproductive Age: A Multilevel Analysis. Asia Pac J Public Health [Internet]. 2020 Dec 8 [cited 2024 Jun 23]; Available from: https://journals.sagepub.com/doi/10.1177/1010539520979924

~~59~~60. Wealth index and other behavioral and sociodemographic characteristics associated with body mass index in Ethiopia - PMC [Internet]. [cited 2024 Jun 23]. Available from: https://www.ncbi.nlm.nih.gov/pmc/articles/PMC8142017/

~~60~~61. Heo J, Yu SY, Yi J, Nam YS, Son DT, Oh J, et al. Wealth gradient-based divergence in the prevalence of underweight among women by marital status in Quoc Oai district, Vietnam. Glob Health Action. 2018 Jan;11(1):1449430.

~~61~~62. Anekwe CV, Jarrell AR, Townsend MJ, Gaudier GI, Hiserodt JM, Cody Stanford F. Socioeconomics of Obesity. Curr Obes Rep. 2020 Sep;9(3):272–9.

~~62~~63. Yussif MT, Morrison AE, Annan RA. 10-year level, trends and socio-demographic disparities of obesity among Ghanaian adults—A systematic review and meta-analysis of observational studies. PLOS Glob Public Health. 2024 Jan 25;4(1):e0002844.

~~63~~64. Adult body weight trends in 27 urban populations of Brazil from 2006 to 2016: A population-based study - PubMed [Internet]. [cited 2024 Jun 24]. Available from: https://pubmed.ncbi.nlm.nih.gov/30840675/

~~64~~65. Rathnayake N, Rathnayake H, Lekamwasam S. Age-Related Trends in Body Composition among Women Aged 20–80 Years: A Cross-Sectional Study. J Obes. 2022 Feb 2;2022:4767793.

~~65~~66. Lima AP de, Nunes AP de OB, Nicoletti CF, Benatti FB. Trend in the Prevalence of Overweight and Obese Adults in São Paulo, Brazil: Analysis between the Years 2006 and 2019. Int J Environ Res Public Health. 2024 Apr;21(4):502.

~~66~~67. Cobb LK, McAdams-DeMarco MA, Gudzune KA, Anderson CAM, Demerath E, Woodward M, et al. Changes in Body Mass Index and Obesity Risk in Married Couples Over 25 Years. Am J Epidemiol. 2016 Mar 1;183(5):435–43.

~~67~~68. Assari S. Education Attainment and Obesity : Differential Returns Based on Sexual Orientation. Behav Sci Basel Switz. 2019 Jan 29;9(2):16.

~~68~~69. Education and obesity at age 40 among American adults - PubMed [Internet]. [cited 2024 Jun 24]. Available from: https://pubmed.ncbi.nlm.nih.gov/23246398/

~~69~~70. Sart G, Bayar Y, Danilina M. Impact of educational attainment and economic globalization on obesity in adult females and males: Empirical evidence from BRICS economies. Front Public Health [Internet]. 2023 Feb 14 [cited 2024 Jun 24];11. Available from: https://www.frontiersin.org/journals/public-health/articles/10.3389/fpubh.2023.1102359/full

~~70~~71. Ethnicity & Disease | JSTOR [Internet]. [cited 2024 Jun 24]. Available from: https://www.jstor.org/journal/ethnicitydisease

~~71~~72. Shiyab W, Halcomb E, Rolls K, Ferguson C. The Impact of Social Media Interventions on Weight Reduction and Physical Activity Improvement Among Healthy Adults: Systematic Review. J Med Internet Res. 2023 Mar 16;25:e38429.

Formatted: Font: +Body (Calibri)

~~72~~73. Internet Use in Relation to Overweight and Obesity: A Systematic Review and Meta-Analysis of Cross-Sectional Studies - PMC [Internet]. [cited 2024 Jun 24]. Available from: https://www.ncbi.nlm.nih.gov/pmc/articles/PMC7442323/

~~73~~74. Liu Q, Li X. The Interactions of Media Use, Obesity, and Suboptimal Health Status: A Nationwide Time-Trend Study in China. Int J Environ Res Public Health. 2021 Dec 15;18(24):13214.

~~74~~75. Loh YL, Yaw QP, Lau Y. Social media-based interventions for adults with obesity and overweight: a meta-analysis and meta-regression. Int J Obes. 2023 Jul;47(7):606–21.

~~75~~76. Stanford FC, Tauqeer Z, Kyle TK. Media and Its Influence on Obesity. Curr Obes Rep. 2018 Jun;7(2):186–92.

~~76~~77. Full article: Obesity and employment as predictors of diabetes in Mexican Americans: findings from a longitudinal study [Internet]. [cited 2024 Jun 24]. Available from: https://www.tandfonline.com/doi/full/10.1080/00036840701335629

~~77~~78. Mesfin BA, Argaw AT, Getahun Negash F, Emiru DA, Aserese AD, Matebe GY. Minimum Dietary Diversity and Associated Factors Among Pregnant Women Living in Arba Minch Health and Demographic Surveillance Sites, Southern Ethiopia, 2022. Health Serv Res Manag Epidemiol. 2023 Jan 1;10:23333928231166671.

~~78~~79. Hamid SBA, Razali MFM, Suhaimi FA, Nor NM. Association of Food Insecurity and Weight Status of Women in Tuba Island, Malaysia. Curr Res Nutr Food Sci J. 2021 Aug 27;9(2):441–9.

~~79~~80. TAGHDIR M, ALIMOHAMADI Y, SEPANDI M, REZAIANZADEH A, ABBASZADEH S, MAHMUD FM. Association between parity and obesity: a cross sectional study on 6,447 Iranian females. J Prev Med Hyg. 2020 Oct 6;61(3):E476–81.

~~80~~81. Sun MH, Wen ZY, Wang R, Gao C, Yin JL, Chang YJ, et al. Parity and Metabolic Syndrome Risk: A Systematic Review and Meta-Analysis of 15 Observational Studies With 62,095 Women. Front Med. 2022 Jul 12;9:926944.

~~81~~82. Abrams B, Heggeseth B, Rehkopf D, Davis E. Parity and body mass index in US women: A prospective 25-year study. Obesity. 2013;21(8):1514–8.

~~82~~83. Endalifer ML, Diress Alen G, Addisu A, Linger B. The association between combined oral contraceptive use and overweight/obesity: a secondary data analysis of the 2016 Ethiopia Demographic and Health Survey. BMJ Open. 2020 Dec;10(12):e039229.

~~83~~84. Simmons KB, Edelman AB. Hormonal contraception and obesity. Fertil Steril. 2016 Nov 1;106(6):1282–8.

~~84~~85. Lopez LM, Bernholc A, Chen M, Grey TW, Otterness C, Westhoff C, et al. Hormonal contraceptives for contraception in overweight or obese women - Lopez, LM - 2016 | Cochrane Library. [cited 2024 Jun 24]; Available from: https://www.cochranelibrary.com/cdsr/doi/10.1002/14651858.CD008452.pub4/full

~~85~~86. Progestin-only contraceptives: effects on weight - Lopez, LM - 2016 | Cochrane Library [Internet]. [cited 2024 Jun 24]. Available from: https://www.cochranelibrary.com/cdsr/doi/10.1002/14651858.CD008815.pub4/references

~~86~~87. Meta-Analysis the Effect of Hormonal Contraception on the Weight Gain and Hypertension in Women of Reproductive Age | Journal of Maternal and Child Health [Internet]. [cited 2024 Jun 24]. Available from: https://thejmch.com/index.php/thejmch/article/view/868

~~87~~88. Kedir H, Berhane Y, Worku A. Magnitude and determinants of malnutrition among pregnant women in eastern Ethiopia: evidence from rural, community-based setting. Matern Child Nutr. 2016 Jan;12(1):51–63.

~~88~~89. Maitra C, Sethi V, Unisa S, Shankar S. Household Food Insecurity and Maternal and Child Nutritional Status: Evidence from Maharashtra. Rev Income Wealth. 2019;65(S1):S63–101.

~~89~~90. Yamada M, Hapsari ED, Matsuo H. Behaviors toward Noncommunicable Diseases Prevention and their Relationship with Physical Health Status among Community-dwelling, Middle-aged and Older women in Indonesia. Int J Environ Res Public Health. 2020 Mar 30;17(7):2332.

~~90~~91. Boutari C, Mantzoros CS. A 2022 update on the epidemiology of obesity and a call to action: as its twin COVID-19 pandemic appears to be receding, the obesity and dysmetabolism pandemic continues to rage on. Metabolism. 2022 Aug;133:155217.

~~91~~92. New UN report: 43.2 million people suffer from hunger in Latin America and the Caribbean, and the region has higher levels of overweight and obesity than the global estimate [Internet]. FAO-RLC. [cited 2024 Jun 25]. Available from: https://www.fao.org/americas/news/news-detail/New-UN-report-43-2-million-people-suffer-from-hunger-in-Latin-America-and-the-Caribbean-and-the-region-has-higher-levels-of-overweight-and-obesity-than-the-global-estimate-/en

~~92~~93. Obesity and overweight populations in Latin America [Internet]. [cited 2024 Jun 25]. Available from: https://www.thelancet.com/campaigns/kidney/updates/obesity-and-overweight-populations-in-latin-america

~~93~~94. Lm J, Mm S, Bm P. Recent trends in the prevalence of under- and overweight among adolescent girls in low- and middle-income countries. Pediatr Obes [Internet]. 2015 Dec [cited 2024 Jun 25];10(6). Available from: https://pubmed.ncbi.nlm.nih.gov/25558987/

~~94~~95. Baruwa OJ, Gbadebo BM, Adeleye OJ, Tabana H, Fagbamigbe AF. Decomposing the rural-urban disparities in overweight and obesity among women of reproductive age in Nigeria. BMC Womens Health. 2023 Dec 21;23(1):680.

~~95~~96. Thapa R, Dahl C, Aung WP, Bjertness E. Urban–rural differences in overweight and obesity among 25–64 years old Myanmar residents: a cross-sectional, nationwide survey. BMJ Open. 2021 Mar 2;11(3):e042561.

~~96~~97. Yeshaw Y, Kebede SA, Liyew AM, Tesema GA, Agegnehu CD, Teshale AB, et al. Determinants of overweight/obesity among reproductive age group women in Ethiopia: multilevel analysis of Ethiopian demographic and health survey. BMJ Open. 2020 Mar 1;10(3):e034963.

Table 1: Socio-economic characteristics of women of reproductive age in 40 low and middle-income countries, 2015 to 2022

Characteristics	Weighted frequency (%)	Underweight (95% CI)	Overweight & obesity (95% CI)
Region			
Africa	203,839 (19.5)	11.2 [11.1-11.3]	27.7 [27.5-27.9]
Asia	796,119 (75.2)	17.0 [16.9-17.1]	23.8 [23.7-23.9]
Others*	44,381 (4.2)	4.8 [4.6-5.0]	4.6 [4.5-4.6]
Place of residence			
Urban	365,235 (35.0)	10.5 [10.4-10.6]	36.7 [36.5-36.8]
Rural	679,104 (56.0)	17.4 [17.3-17.5]	20.7 [20.6-20.8]
Covered by health insurance (n = 993,367)			
No	738,086 (74.3)	15.1 [15.0-15.2]	26.6 [25.5-25.7]
Yes	255,281 (25.7)	15.9 [15.7-16.0]	25.3 [25.1-25.5]
Household wealth			
Poor	397,860 (38.1)	19.9 [19.7-20.0]	16.4 [16.2-16.5]
Middle	213,202 (20.4)	14.8 [14.6-14.9]	25.3 [25.1-25.5]
Rich	433,278 (41.5)	10.6 [10.5-10.7]	35.7 [35.6-35.9]
Age			
15-25 year	357,587 (34.2)	25.0 [24.9-25.2]	11.0 [10.9-11.1]
25-34 year	319,714 (30.6)	12.0 [11.9-12.1]	27.7 [27.5-27.8]
>=35 years	367,038 (35.2)	8.9 [8.8-9.0]	37.6 [37.4-37.7]
Educational status			
No formal education	237,726 (22.8)	15.9 [15.8-16.1]	21.3 [21.2-21.5]
Primary education	185,877 (17.8)	12.4 [12.2-12.5]	28.7 [28.5-28.9]
Secondary education	480,398 (46.0)	17.1 [17.0-17.2]	24.9 [24.7-25.0]
Higher education	140,336 (13.4)	11.9 [11.7-12.1]	31.0 [30.7-31.3]
Working status (n = 455,170)			
Non-working	252,205 (55.4)	13.9 [13.7-14.0]	28.4 [28.3-28.6]
Working	202,965 (44.6)	10.0 [9.9-10.1]	31.8 [31.6-32.0]
Marital status			
Never in union	254,214 (24.3)	26.3 [26.1-26.5]	11.3 [11.2-11.4]
Currently in union	733,430 (70.2)	11.7 [11.6-11.8]	30.0 [29.9-30.1]
Formerly in union	56,695 (5.5)	11.1 [10.9-11.4]	32.6 [32.2-33.0]
Media exposure			
No	446,154 (42.7)	18.1 [18.0-18.2]	18.5 [18.4-18.7]

Yes	598,186 (57.3)	13.1 [13.0-13.2]	31.1 [31.0-31.2]
Household head			
Male	839,056 (80.3)	15.6 [15.5-15.6]	25.0 [24.9-25.1]
Female	205,284 (19.7)	14.5 [14.3-14.6]	27.3 [27.0-27.4]

832 ***Others**: Latin America, Caribbean, and Europe.

Table 2: Reproductive and health-related characteristics of women of reproductive age in 40 low and middle-income countries, 2015 to 2022

**Characteristics**	**Weighted frequency (%)**	**Underweight (95% CI)**	**Overweight & obesity (95% CI)**
Age at marriage (n =790,125)			
< 18 year	329,519 (41.7)	13.2 [13.1-13.4]	27.3 [27.2-27.5]
>= 18 year	460,606 (58.3)	10.6 [10.6-10.7]	32.1 [32.0-32.2]
History of pregnancy loss			
No	907,456 (86.9)	16.1 [16.0-16.2]	23.8 [23.7-23.9]
Yes	136,858 (13.1)	10.1 [9.9-10.3]	37.2 [36.9-37.4]
Parity			
Nullipara	310,076 (29.7)	24.7 [24.5-24.8]	12.7 [12.6-12.9]
Primipara	151,396 (14.5)	12.7 [12.5-12.8]	26.9 [26.6-27.1]
Multipara	479,261 (45.9)	10.8 [10.7-10.9]	32.4 [32.2-32.6]
Grand-multipara	103,607 (9.9)	11.8 [11.7-12.0]	30.2 [30.0-30.5]
Number of U5 children			
0	587,244 (56.2)	15.8 [15.7-15.9]	26.3 [26.2-26.4]
1	266,948 (25.6)	14.2 [14.1-14.3]	26.2 [26.0-26.3]
2+	190,148 (18.2)	15.6 [15.4-15.7]	21.8 [21.6-22.0]
Birth order			
1 to 3	548,478 (52.5)	11.2 [11.1-11.3]	31.1 [30.9-31.2]
>=4	495,862 (47.4)	19.8 [19.6-19.9]	19.6 [19.5-19.7]
Preceding birth interval (n = 580,547)			
< 33 months	274,304 (47.2)	12.1 [11.9-12.2]	29.3 [29.1-29.5]
>= 33 months	306,243 (52.8)	10.0 [9.9-10.1]	34.4 [34.3-34.6]
Anemia status (n = 951,070)			
No anemic	457,754 (48.1)	12.9 [12.8-13.0]	28.3 [28.2-28.5]
Anemia	493,316 (51.9)	18.1 [17.9-18.2]	21.7 [21.6-21.8]
Contraceptive use			
Not using	584,733 (56.0)	18.4 [18.3-18.5]	21.2 [21.1-21.3]

Using	459,607 (44.0)	11.2 [11.1-11.3]	31.2 [31.0-31.3]
Perceived distance to health facility (1,026,269)			
Not a big problem	752,281 (73.3)	14.8 [14.7-14.9]	27.0 [26.9-27.1]
Big problem	273,988 (26.7)	16.8 [16.6-16.9]	27.1[21.4-21.7]
Decision maker on women’s health			
Woman alone	57,115 (5.5)	7.8 [7.6-8.0]	40.0 [39.6-40.4]
Jointly with partner	164,450 (15.7)	9.4 [9.3-9.6]	36.1 [35.8-36.3]
Others*	822,774 (78.8)	17.1 [17.0-17.1]	22.3 [22.3-22.4]
Toilet facility			
Unimproved	290,319 (27.8)	19.8 [19.6-19.9]	16.8 [16.7-16.9]
Improved	754,021 (72.2)	13.7 [13.6-13.7]	28.8 [28.7-28.9]
Drinking water source			
Unimproved	145,724 (14.0)	15.4 [15.2-15.5]	21.4 [21.2-21.6]
Improved	898,615 (86.0)	15.4 [15.3-15.4]	26.2 [26.1- 26.3]

*Others: husband only; someone else; other DHS category.

Table 3: Country-specific prevalence of underweight and overweight and obesity among women of reproductive age in 40 low- and middle-income countries, 2015 to 2022

Country	Survey year	Underweight	Overweight	Obesity	Total
Albania	2018	463 (4.3)	3108 (29.1)	1755 (16.4)	10668
Armenia	2016	210 (3.6)	1755 (29.9)	883 (15.0)	5877
Bangladesh	2018	2285 (11.5)	5037 (25.5)	1291 (6.5)	19786
Burkina Faso	2021	1030 (11.6)	1401 (15.8)	522 (5.9)	8852
Benin	2018	815 (10.1)	1362 (16.9)	732 (9.1)	8065
Burundi	2017	1520 (17.6)	575 (6.7)	153 (1.8)	8620
Cote d'Ivoire	2021	533 (7.4)	1533 (21.2)	867 (12.0)	7214
Cameroon	2018	395 (5.7)	1648 (23.9)	927 (13.4)	6892
Ethiopia	2016	3188 (21.1)	955 (6.3)	229 (1.5)	15123
Gabon	2019	458 (7.5)	1451 (23.8)	1517 (24.8)	6111
Ghana	2022	544 (7.0)	1917 (24.8)	1447 (18.7)	7724
Gambia	2019	762 (12.8)	1344 (22.6)	810 (13.6)	5952
Guinea	2021	480 (9.0)	970 (18.2)	434 (8.1)	5322
Guatemala	2015	688 (2.9)	7720 (31.9)	4813 (19.9)	24184
Haiti	2016	966 (10.1)	1998 (21.0)	1076 (11.3)	9530
India	2021	127250 (18.4)	121664 (17.6)	43643 (6.3)	692238
Jordan	2017	94 (1.3)	2722 (38.0)	2189 (30.6)	7155

Kenya	2022	1513 (9.2)	3995 (24.2)	2322 (14.1)	16486
Cambodia	2022	1024 (10.4)	2241 (22.8)	570 (5.8)	9833
Liberia	2019	208 (5.1)	1012 (24.8)	501 (12.3)	4083
Madagascar	2021	1680 (17.6)	1077 (11.3)	262 (2.7)	9553
Mali	2018	480 (9.4)	994 (19.4)	422 (8.2)	5119
Myanmar	2015	1898 (15.0)	2428 (19.2)	705 (5.6)	12640
Mauritania	2019	538 (7.3)	1980 (26.9)	1962 (26.6)	7363
Maldives	2016	724 (10.3)	2112 (30.2)	1373 (19.6)	6998
Malawi	2015	534 (6.7)	1284 (16.0)	429 (5.4)	8009
Mozambique	2022	398 (6.7)	953 (16.0)	452 (7.6)	5955
Nigeria	2018	1672 (11.3)	2703 (18.4)	1442 (9.8)	14730
Nepal	2022	961 (13.0)	1645 (22.3)	509 (6.9)	7370
Pakistan	2017	350 (8.0)	1329 (30.2)	925 (21.0)	4400
Rwanda	2019	405 (5.6)	1559 (21.5)	432 (6.0)	7265
Sierra Leone	2019	486 (6.5)	1517 (20.1)	599 (8.0)	7530
Chad	2015	2029 (17.9)	1085 (9.6)	263 (2.3)	11355
Tajikistan	2017	744 (7.0)	2597 (24.3)	1386 (13.0)	10669
Timor-Leste	2016	3180 (25.5)	1076 (8.6)	204 (1.6)	12451
Turkey	2018	250 (3.7)	1977 (29.5)	2026 (30.2)	6701
Tanzania	2022	696 (9.2)	1465 (19.4)	873 (11.5)	7570
Uganda	2016	481 (8.0)	1036 (17.2)	428 (7.1)	6012
Zambia	2018	97 (3.0)	875 (26.9)	1150 (35.4)	3251
Zimbabwe	2015	554 (5.7)	2251 (23.2)	1195 (12.3)	9684

Table 4: Measures of variations and model statistics summary for determinants of ~~abnormal nutritional indices~~underweight and overweight and obesity among women of reproductive age in 40 LMICs, 2015 to 2022

**Measure of variation**	**Model 1**	**Model 2**	**Model 3**	**Model 4**
Cluster-level variance	0.44	0.27	0.23	31
Intra-class correlation	0.26	0.28	0.17	0.22
Explained variance (PCV)	Reference	0.39	0.47	0.29
Median odds ratio	1.72	1.35	1.24	1.44
**Model statistics summary**				

Log-likelihood	-242,243	-156,670	-242,128	-156,148
Deviance	484,486	313,340	482,256	312,296

Table 5: Determinants of ~~abnormal nutritional indices~~underweight and overweight and obesity among women of reproductive age in 40 low and middle-income countries, 2015 to 2022

**Characteristics**	**Underweight ARRR (95% CI)**	**Overweight and Obesity ARRR (95% CI)**
Household wealth		
Poor	Ref	Ref
Middle	0.80 (0.78, 0.83)*	1.31 (1.29, 1.34)**
Rich	0.69 (0.67, 0.71)*	1.64 (1.61, 1.67)**
Age at marriage		
< 18 year	Ref	Ref
>= 18 year	0.93 (0.91-0.96)*	0.96 (0.94, 0.97)**
Age		
15-25 year	Ref	Ref
25-34 year	0.79 (0.77, 0.82)*	2.11 (2.05, 2.17)*
>=35 years	0.73 (0.70, 0.75)*	3.64 (3.53, 3.74)*
Educational status		
No formal education	Ref	Ref
Primary education	0.68 (0.66, 0.71)*	1.56 (1.52, 1.59)**
Higher education	0.65 (0.63, 0.67)*	1.91 (1.87, 1.96)**
Access to media		
No	Ref	Ref
Yes	0.79 (0.77, 0.81)*	1.60 (1.57, 1.63)**
Employment status		
Non-working	Ref	Ref
Working	0.89 (0.87, 0.91)*	0.85 (0.84, 0.86)*
Parity		
Nullipara	Ref	Ref
Primipara	0.99 (0.95, 1.04)	0.93 (0.90, 0.96)
Multipara	0.94 (0.89, 0.98)*	1.03 (0.99, 1.07)
Contraceptive use		
Not using	Ref	Ref
Using	0.94 (0.92, 0.97)*	1.22 (1.20, 1.25)**

Decision maker on women's health		
Woman alone	Ref	Ref
Jointly with partner	1.09 (1.05, 1.13)*	0.94 (0.92, 0.96)*
Others[+]	1.08 (1.04, 1.12)*	0.86 (0.84, 0.88)*
Region		
Africa	Ref	Ref
Asia	1.46 (1.42, 1.50)*	1.00 (0.98, 1.02)
Others[++]	0.40 (0.37, 0.43)*	2.27 (2.20, 2.33)**
Place of residence		
Urban	0.80 (0.77, 0.83)*	1.73 (1.70, 1.76)*
Rural	Ref	Ref

[+]Others: husband only; someone else; other DHS category. [++]Others: Latin America, Caribbean, and Europe. *P-value<0.05. **P-value<0.01. "Normal Weight" was the base category.

S1. STROBE Statement—checklist of items that should be included in reports of observational studies.

Dear Academic Editor,

Thank you again for the opportunity to revise our manuscript "***Level of overweight and obesity surpassed underweight among women in 40 low and middle-income countries: findings from a multilevel multinomial analysis of population survey data (ID: PONE-D-24-26578R1)***". We have addressed the concerns raised using a point-by-point response as stated below. The amendments made to the manuscript have been presented using track change in the attachment titled "Revised manuscript with track changes".

Response to Editor's comments

Comment 1: The Methods Section casts doubts on the estimate: (i) It appears that authors are referring to many dependent variables yet there is only one (BMI, categorized); (ii) It is unclear in this section that overweight and obesity are treated as one category; (iii) while authors are doing multilevel modeling, it is unclear in this section what are Levl-1 and Level-2 variables. More importantly, it is unclear how Level-2 variables (also referred to as community variables) were constructed.

Response 1: Thank you for your important concern and sorry for the editorial errors. We have amended the description of the dependent variable (BMI) and clarified that overweight and obesity were treated as one category. Additionally, we have specified level-1 and level-2 variables in the revised manuscript.

Comment 2: It is unclear that analyses were performed on sub-samples of women of reproductive ages in all surveys since DHSs don't collect weight and height for all women to compute BMI. As such, authors took the freedom to DROP cases with missing information on BMI. This is a "big mistake from the sampling design point of view" because it will produce biased estimates. (see https://stats.oarc.ucla.edu/stata/faq/how-can-i-analyze-a-subpopulation-of-my-survey-data-in stata/#:~:text=As%20stated%20in%20the%20Stata,are%20excluded%20from%20the%20analysis.)

Response 2: Thank you very much for pointing out this. We have stated that the analysis was performed on the sub-sample of women of reproductive age in all surveys. Regarding missing

information, we agree with your concern that missing data can result in a biased estimate if not properly handled. For this reason, we have handled the missing data based on DHS guidelines (i.e. "women who were not weighed and measured and women whose values for weight and height were not recorded are excluded from both the denominator and the numerators.") (see https://www.dhsprogram.com/pubs/pdf/DHSG1/Guide_to_DHS_Statistics_DHS-8.pdf on Page 11.10). Additionally, we have also acknowledged the drawback of missing data in the limitation section of the revision.

Comment 3: Finally, the Discussion Section also needs refinement because it is redundant with findings. The authors need to engage in discussion with previous studies on the same topic while highlighting regional differences and implications for future health interventions.

Response 3: Thank you for your important recommendation. We have removed redundant results throughout the discussion section as per your suggestion.

Response to comments in the annotated manuscript

Comment 1: Line 22-24: A methodological challenge arises when pooling data. I will look into the Methods Section but this needs to be clear on how Complex Survey Design was implemented in pooled data.

Response 1: Thank you for your concern. As we have stated in the data management and analysis sub-heading of the method section that we have used pooled/appended demographic and health survey data from 44 countries for the current analysis.

Comment 2: Line 26: abnormal nutritional indices (This means what?)

Response 2: Thank you. It was to indicate malnutrition (i.e. underweight and overweight and obesity). As a result, we have specified this concern in the revised manuscript.

Comment 3: Line 30-31: Now and again, this is the pitfall I usually see in pooled data since LMICs are not a homogeneous group on the one hand, and context matters on the other hand. Is this finding informative?

Response 3: Thank you very much for your concern. We have carried out a subgroup analysis to show regional differences in the revised manuscript. We also agree that pooling data from

different countries has its drawbacks since the countries are not homogeneous and survey years are different. For this reason, we have considered these issues under the limitation section.

Comment 4: Line 33: women of middle and upper-class families?

Response 4: Thank you and sorry for the grammar error. We have changed this phrase into "women of families with middle and rich wealth indexes" accordingly in the revised manuscript.

Comment 5: Line 34: From primary to higher education? What about those with secondary education?

Response 5: Thank you for your question. For the analysis purpose, we have initially categorized educational status as no formal education, primary, secondary, and higher education. However, when we fit the regression model, the model omits the estimation of the relative risk ratio for the category "secondary education". As a result, we recategorized this variable as "no formal education", "primary education", and "higher education (i.e. secondary + higher)".

Comment 6: Line 37: You need to clarify the difference between middle- and upper-income families (By the way, income is not captured in DHS), and rich households.

Response 6: Thank you for your suggestion. In DHS, the household wealth index is categorized as poorest, poorer, middle, richer, and richest. For the analysis purpose, we have recategorized it as "poor", "middle", and "rich" based on the literature. Based on your suggestion, we changed these phrases to their correct DHS categories.

Comment 7: Line 43: modifiable risk factors of what?

Response 7: Thank you. It is just to indicate modifiable risk factors of both undernutrition and overnutrition. We have clarified this concern in the revised manuscript.

Comment 8: Line 62-64: You have furthermore and additionally in the same paragraph?

Response 8: Thank you very much. We have addressed this concern accordingly in the revised manuscript.

Comment 9: Line 72-74: You cannot list figures like this. Rewrite the sentence... using for instance: ranging from .... to ....

Response 9: Thank you for your interesting suggestion. We have revised this sentence as per your recommendation.

Comment 10: Line 77-80: same as above comment.
Response 10: Thank you for your interesting suggestion. We have revised this sentence as per your recommendation.

Comment 11: Line 103: Again, this poses a methodological concern that needs further scrutiny for reported findings to be trusted. 1) In the DHS, not all women in surveys were measured (weight, height) to compute BMI. As such, only a sub-sample of women has valid information on BMI. 2) How the complex survey design of DHS was treated in this case?
Response 11: Thank you for your important concern. Indeed, all women in the survey do not have BMI data (missing or not measured). For this reason, we have excluded cases with missing data based on DHS guidelines for handling missing data, and this issue was also acknowledged in the limitation section. Regarding the treatment complex survey design, we have used a multilevel model, which accounts for the hierarchical nature of the complex survey.

Comment 12: Line 107: This is not TRUE. The dependent variable is only one: BMI. Analytically, it was grouped into four categories: 1) underweight; 2) normal weight; 3) overweight; and 4) obese. Now, in many developing countries, obesity might represent a marginal percentage, which could lead to unstable statistical models. Please clarify your line of thought.
Response 12: Thank you very much for pointing out this important issue. As per your recommendation, we have clarified the dependent variable as BMI, with its respective categories (underweight, Normal, and overweight and obesity). We have also stated that overweight and obesity are treated as one category in the revised manuscript.

Comment 13: Line 112- Since you applied multilevel models, it helps readers if variables are grouped for each level
Response 13: Thank you for your suggestion. We have specified level-1 and level-2 variables in the revision.

Comment 14: Line 128-129: This is a serious methodological pitfall since it alters the survey design and produces "flawed" estimates

Response 14: Thank you very much for pointing out this. We have stated that the analysis was performed on the sub-sample of women of reproductive age from all surveys in the revision. Regarding missing information, we agree with your concern that missing data can result in a biased estimate if not properly handled. For this reason, we have handled the missing data based on DHS guidelines (i.e. "women who were not weighed and measured and women whose values for weight and height were not recorded are excluded from both the denominator and the numerators.") (see https://www.dhsprogram.com/pubs/pdf/DHSG1/Guide_to_DHS_Statistics_DHS-8.pdf on Page 11.10). Additionally, we have also acknowledged the drawback of missing data in the limitation section of the revision.

Comment 15: Line 132: Level 1? Level 2? In short, how many LEVELS?

Response 15: Thank you very much. We have specified level-1 and level-2 variables under the "Independent variables" subsection of the revised manuscript.

Comment 16: Line 46: unknown as presented

Response 16: Thank you for your concern. We have replaced this with level-1 variables as per your suggestion in the "Independent variables" section.

Comment 17: Line 181: full term of WRA.

Response 17: Thank you. We have provided the extended form of WRA (i.e. women of reproductive age) in the revised result section.

Comment 18: Line 192: Reproductive and health-related characteristics. This appears for the first time.

Response 18: Thank you for your concern. For the descriptive purpose, we have separately presented the characteristics of the study participants as "Sociodemographic and reproductive-related characteristics".

Comment 19: Line 211: In Figures 1-2, countries are thrown as they belonged to the same geographic region. Groupings need to be done.

Response 19: Thank you for your interesting comment. We have carried out subgroup analysis to show country-, regional, and overall estimates of underweight and overweight and obesity based on your suggestion. As a result, we have changed Figures 1 and 2 based on the reanalysis results.

Comment 20: Line 214: Finally, this contradicts what is said in the Methods Section regarding BMI (4 categories) but findings use only categories

Response 20: Thank you for your concern. We have modified the definition and categories of the outcome variable and stated that overweight and obesity are treated as one category as per our suggestion.

Comment 21: Line 266: As it stands now, the discussion is flawed: It is mostly a repetition of findings.

Response 21: Thank you for your important recommendation. We have removed redundant results throughout the discussion section as per your suggestion.

Comment 22: Line 267: It should be clear throughout the manuscript that overweight and obesity are treated as one category.

Response 22: Thank you for your suggestion. We have clarified that overweight and obesity were treated as one category throughout the revised manuscript.

Comment 23: Line 292: what does middle class mean? You use interchangeably income, class, households???? WHY, explain!

Response 23: Thank you very much and sorry for the editorial error. We have consistently used the appropriate terms for the household wealth index throughout the revised manuscript.

Comment 24: Line 313: what about women with secondary education

Response 24: Thank you for your question. For the analysis purpose, we have initially categorized educational status as no formal education, primary, secondary, and higher education. However, when we fit the regression model, the model omits the estimation of the relative risk ratio for the category “secondary education”. As a result, we re-categorized this variable as “no formal education”, “primary education”, and “higher education (i.e. secondary + higher)”.

Comment 25: Line 318-319: I expect that higher education leads to wealth, but at the same time leads to sedentary lifestyles (always in cars, with maids in houses, low levels of physical activity).
Response 25: Thank you for your interesting comment. We have added this explanation to the revised manuscript.

Comment 26: Line 346: Please be specific
Response 26: Thank you. We have amended this concern throughout the revised manuscript.

Response to Reviewers' comments

Reviewer #1: The manuscript entitled "Level of overweight and obesity surpassed underweight among women in 40 low and middle-income countries: findings from a multilevel multinomial analysis of population survey data". I appreciate the authors' efforts. The authors manage to respond to all the issues perfectly.
Response: Thank you for your appreciation and for accepting our manuscript in its current form.

Reviewer #2: The authors addressed all the comments and concerns raised in the first revision. I have no further comment.
Response: Thank you for accepting our manuscript in its current form.
