# Supplementary material for: Level of overweight and obesity surpassed underweight among women in 40 low and middle-income countries: Findings from a multilevel multinomial analysis of population survey data
Source: PLoS One. 2025 Mar 26;20(3):e0320095. doi: 10.1371/journal.pone.0320095 (PMC11940715; doi:10.1371/journal.pone.0320095)
Supplement: Attachment — Submitted filename: Response to Reviewers.docx [file pone.0320095.s004.docx]

**Dear Academic Editor**,

Thank you for the opportunity to revise our manuscript. “Level of overweight and obesity surpassed underweight among women in 40 low and middle-income countries: findings from a multilevel multinomial analysis of population survey data (ID: PONE-D-24-26578)”. We have addressed the concerns raised by the reviewers using a point-by-point response as stated below. The amendments made to the manuscript have been presented using track change in the attachment titled “Revised manuscript with track changes”.

**Response to Reviewer #1 Comments**

**Comment 1:** This article provides advanced information on the prevalence of overweight and obesity among women in 40 low- and middle-income countries exceeded underweight. This manuscript should go the following suggested revision for improvement. Many sentences were started using gerund, authors are suggested to rewrite using simple or compound format. English should be checked by professionals

**Response 1:** Thank you for your suggestion. We have thoroughly revised the manuscript for grammar and editorial errors.

**Comment 2:** L17, L20, L23, Use the elaborate form of NCD, LMIC,, DHS

**Response 2:** Thank you very much for your suggestion. We have provided the elaborated form for the stated abbreviations in the revised manuscript.

**Comment 3:** L28, IC to CI

**Response 3:** Thank you and sorry for the editorial error. We have changed IC to CI.

**Comment 4:** L33, Add more core results such as women’s education, age, employment status, contraceptive use, region, place of residence, and media access on the risk of being underweight, overweight, and obese. If authors have no room, contract the conclusion section (L34-41) and add the above-mentioned results as much as possible.

**Response 4:** Thank you for your interesting comment. We have added the core results as per your suggestion in the abstract section of the revised manuscript.

**Comment 5:** L48, deposits to deposition

**Response 5:** Thank you very much. We have changed this in the revised manuscript.

**Comment 6:** L49-51, rewrite as, There are several linkages between obesity and health problems, including type 2 diabetes and heart disease, poor bone health, impaired reproductive function, and cancers of the reproductive organs [1,3,4].

**Response 6:** Thank you for your suggestion. We have incorporated the recommended change into the revised manuscript.

**Comment 7:** L77, World health organization (WHO)

**Response 7:** Thank you. We have considered the suggested change in the revision.

**Comment 8:** L91, add sources (link/links or state the organization name of collector).

**Response 8:** Thank you for your recommendation. We have provided a web link for the data source as suggested.

**Comment 9:** L168, end of the sentence (Table 1).

**Response 9:** Thank you. We have cited the table as per your recommendation.

**Comment 10:** L174-175, L188-189, L210-211, delete. It is better not to include in the main text body. It has already been cited in the main text and given in the end of the manuscript which cause repetition and similar comments applies for figures captions in the main text body.

**Response 10:** Thank you. We agree with your concern, but the journal guideline for preparing tables and figures suggests placing their captions in the main body of the manuscript as well as with each table and figure at the end of the main body.

**Comment 11:** L187, Includes an explanation of other parameters which was stated in Table 2.

**Response 11:** Thank you very much. We have added the explanation for other parameters as per your recommendation.

**Comment 12:** L298, Add a more concise form of the result section.

**Response 12:** Thank you. We have modified this section as suggested.

**Comment 13:** L318, cite table of figure number according to your statement.

**Response 13:** Thank you very much. We have cited the table and figure accordingly in the revision.

**Comment 14:** L723, Use different “*” or symbols to show different levels of significance values. For instance, * for 0.0.5, ** for 0.01

**Response 14:** Thank you. We have checked the regression table and provided a respective symbol to indicate different levels of significance as suggested in the revision.

**Comment 15:** Figure 3, vertical axis, Percent (%) to Percentage (%). Figure 3 has a similar trend in 2015 and 2022 for each parameter. Explain this phenomena in the result section (L220).

**Response 15:** Thank you for your suggestion. We have changed percent to percentage and explained the result observed between 2015 and 2022 in the revised manuscript.

**Response to Reviewer #2 Comments**

This is a well-written manuscript assessing abnormal nutritional indices, which describes the analysis of demographic and health survey data from 40 low- and middle-income countries utilizing a large sample size.

**Comment 1:** Line number 86, please consider changing ‘reproductive age group women’ to ‘women of reproductive age’ (WRA) and replacing ‘women’ with ‘WRA’.

**Response 1:** Thank you for your recommendation. We have made this change throughout the manuscript where it is appropriate.

**Comment 2:** Why are your study population women of reproductive age? What is the rationale?

**Response 2:** Thank you for your important concern. As we know, the Demographic and Health Survey (DHS) collects basic health and health-related information from women, men, and children in the sampled households. For the current analysis, we utilized the women's dataset, focusing on women of reproductive age (WRA), specifically those aged 15 to 49 years, within the households. Therefore, since we analyzed secondary data obtained from this source, the study population consisted of women of reproductive age.

**Comment 3:** Citation is needed for the dependent variable measurement

**Response 3:** Thank you very much. We have provided citation for the measurement of the dependent variable as suggested.

**Comment 4:** How was the variable ‘media exposure’ constructed?

**Response 4:** Thank you for your concern. Media exposure was generated using the frequency of watching television, listening to the radio, and reading newspapers, which have three response options (i.e. not at all, less than once a week, and at least once a week). Using the DHS definition, women who indicated that they watched television, listened to the radio, or read the newspaper at least once a week were classified as having media exposure, while those who did not meet this criterion were classified as not having media exposure. We have added this information under the “Variable and Measurement” section of the revised manuscript.

**Comment 5:** Indicate the outcome variable's reference category in Table 5. It looks like that "Normal Weight" was the base category.

**Response 5:** Thank you. We have indicated the base category of the outcome variable under Table 5 of the revision.

**Comment 6:** It is important to recognize the limitations of BMI in the strengths and limitations section, as it does not differentiate excess fat, muscle, or bone mass.

**Response 6:** Thank you for your important suggestion. We have added this limitation in the revised manuscript.
