# Supplementary material for: Level of overweight and obesity surpassed underweight among women in 40 low and middle-income countries: Findings from a multilevel multinomial analysis of population survey data
Source: PLoS One. 2025 Mar 26;20(3):e0320095. doi: 10.1371/journal.pone.0320095 (PMC11940715; doi:10.1371/journal.pone.0320095)
Supplement: Attachment — Submitted filename: Response to Reviewer.docx [file pone.0320095.s007.docx]

Dear Academic Editor,

Thank you again for the opportunity to revise our manuscript “***Level of overweight and obesity surpassed underweight among women in 40 low and middle-income countries: findings from a multilevel multinomial analysis of population survey data (ID: PONE-D-24-26578R2)***”. We have addressed the concerns raised using a point-by-point response as stated below. The amendments made to the manuscript have been presented using track change in the attachment titled “Revised manuscript with track changes”.

**Response to Editor’s Comments**

**Comment 1:** The presentation of independent variables is still unsatisfactory, and dependent variable as well. If overweight and obesity are treated as one category, this should be done in ALL Tables through the manuscript.

**Response 1:** Thank you very much. We have corrected the presentations of independent and dependent variables as per your suggestions throughout all tables in the revision.

**Comment 2:** The structure of the variables (Level-1) should follow the (i) women-; and (ii) household- variables in this order

**Response 2:** Thank you for your recommendation. We have presented the level-1 variables as per the suggested order in the revised tables.

**Comment 3:** The justification of recording women's education in two categories (no education and higher) is less convincing. I suspect there is a coding inconsistency across countries leading to the error the authors are getting.

**Response 3:** Thank you very much for your interesting recommendation and sorry for providing a misleading justification in the previous response. We have checked the coding of educational status for all countries as suggested. Based on this, we did reanalysis and revised the results and discussion sections accordingly. We appreciate you for this important critique.

**Journal Requirements:**

**Comment:** Please review your reference list to ensure that it is complete and correct. If you have cited papers that have been retracted, please include the rationale for doing so in the manuscript text, or remove these references and replace them with relevant current references. Any changes to the reference list should be mentioned in the rebuttal letter that accompanies your revised manuscript. If you need to cite a retracted article, indicate the article’s retracted status in the References list and also include a citation and full reference for the retraction notice.

**Response:** Thank you. We have reviewed the reference list and confirm that it is complete and correct and no retracted article has been included in the list.
